# Eye movement kinematics reveal novel circadian organization of sleep substates

**DOI:** 10.1038/s41467-026-72222-0

**Published:** 2026-05-05

**Authors:** Vikash Choudhary, Charles R. Heller, Sophie Aimon, Lílian de Sardenberg Schmid, Jennifer M. Li, Drew N. Robson

**Affiliations:** Max Planck Institute for Biological Cybernetics, Tübingen, Germany

**Keywords:** Circadian rhythms and sleep, Neural circuits

## Abstract

In most non-mammalian model organisms, sleep is operationally defined as persistent locomotor quiescence (e.g., ≥1 min) associated with decreased arousal^1–3^. In contrast to the long-established subdivision of mammalian sleep by eye movements^4–9^, the existence of sleep-associated eye movements and, more broadly, discrete sleep substates in non-mammalian organisms remains actively debated^10–14^. Here we present the first systematic investigation of fish eye movements during naturally occurring sleep across the full circadian cycle, under light–dark cycles as well as constant light and constant darkness. Across *Danio* species (*Danio rerio*, *Danio nigrofasciatus* and *Danio aesculapii*), we identify four discrete, conserved sleep substates with circadian organization: three sleep states with distinct eye-movement kinematics (QEM-1, QEM-2 and QEM-3) and one sleep state with no eye movements (QNEM). QNEM predominates at night, QEM-2 increases toward morning, and unexpectedly, QEM-1 occurs almost exclusively during the day. QEM-1 fulfills multiple criteria for sleep in zebrafish, including elevated arousal thresholds, partial loss of postural control, homeostatic rebound after deprivation, noradrenergic suppression, and brain-wide neural dynamics that encode state progression. Altogether, these findings uncover a previously unrecognized sleep architecture in larval fish, in which multiple substates with distinct eye-movement kinematics are conserved across *Danio* species and gated by circadian time and ambient light.

## Abstract (web summary)

Eye movements historically partitioned mammalian sleep into two substates. Here, the authors uncover four sleep substates in fish, each with distinct eye movement kinematics, revealing an intricate sleep architecture gated by circadian time and luminosity.

## Introduction

Across the animal kingdom, behavioral quiescence serves vital biological functions including energy conservation, cognitive processing, memory consolidation, and synaptic homeostasis^6,15–20^. In mammals, locomotor quiescence encompasses multiple distinct sleep states, which are uniquely identified based on a set of neural and behavioral features such as arousal thresholds, muscle tone, cortical activity patterns, and eye movements^4,5,7,9^. In non-mammalian species such as Drosophila and zebrafish, periods of prolonged immobility (e.g., ≥ 1 min) are associated with increased arousal thresholds and operationally defined as sleep. However, recent studies across non-mammalian species have suggested a more complex organizational structure of sleep. Optogenetic activation of the dorsal fan-shaped body in Drosophila induces a paradoxical state where the arousal threshold is significantly increased but brain activity remains at similar levels to wakefulness^21^. Spiders occasionally exhibit spontaneous retinal movements during nighttime quiescence^12^, though the arousal threshold has not yet been quantified in these states. Bearded dragons and octopuses both alternate between multiple sleep states, each with distinct neural and behavioral signatures (e.g., eye movements or skin coloration)^11,22^. Collectively, these findings indicate that for at least some non-mammalian species, sleep is not a monolithic state.

Over the last two decades, the larval zebrafish has become a powerful model for the study of persistent behavioral states, including exploration/exploitation states during foraging, alternating turn states, as well as sleep and wake^3,23–32^. Zebrafish sleep is defined as locomotor quiescent periods lasting ≥ 1 min that are associated with increased arousal thresholds^3,23,26,29,33^. However, whether zebrafish sleep is a monolithic state or can be subdivided based on additional behavioral features has not yet been established. One recent zebrafish sleep study observed distinct neural signatures during locomotor quiescence across 120 min of brain recording, but only after continuous sleep deprivation lasting several days^13^. Whether these distinct neural signatures exist within naturally occurring sleep states remains unknown. Furthermore, the study found no overt behavioral signatures associated with distinct sleep substates.

Intriguingly, previous zebrafish studies have also noted eye movements during brief periods of immobility^34,35^. However, these studies focused either on short-lived immobility states (24.4 ± 2.5 s) triggered by behavioral futility^34^ or the kinematics of isolated eye movements^35^. Arousal thresholds were not systematically characterized in these studies, and the eye movements were not tracked over extended periods of time (e.g., hours or days). Thus, a more systematic understanding of the diversity and organization of naturally occurring locomotor quiescence and sleep across the circadian cycle has yet to be established.

To systematically investigate eye movements during naturally occurring sleep over circadian timescales, we developed a large-scale high resolution (50 μm/pixel) behavioral imaging platform to monitor freely swimming larvae (up to 20 fish per experiment) continuously over multiple days. As expected, across both day and night, zebrafish larvae exhibited extended locomotor quiescence periods (lasting ≥1 min) that are operationally defined as sleep in previous studies^3,30,33,36,37^. Strikingly, joint analysis of locomotion and eye movements reveals four discrete sleep substates – three states with distinct eye-movement kinematics (QEM-1–3) and one state with no eye movements (QNEM). All four states were associated with decreased arousal, fulfilling one of the key criteria of zebrafish sleep.

Notably, these states occupy distinct circadian phases. QNEM and QEM-3 are enriched at night, QEM-2 rises toward morning, and QEM-1 is expressed predominantly during the day, revealing a previously unrecognized organization of larval fish sleep across time of day. Despite occurring during daylight, QEM-1 shows multiple signatures of sleep, including elevated arousal thresholds, partial loss of postural control, homeostatic rebound after deprivation, and noradrenergic suppression. Brain-wide imaging during QEM-1 further reveals a small population of state-encoding neurons that evolve along organized, committed trajectories and track state progression in time. These neural and behavioral observations suggest that the zebrafish brain spontaneously enters a committed sleep state throughout the circadian day.

Importantly, this multi-state sleep architecture uncovered in zebrafish generalizes beyond a single strain or species. The same set of substates and their phase relationship are preserved across zebrafish strains and across closely related *Danio* species, including *D. nigrofasciatus* and *D. aesculapii*. Moreover, constant-light and constant-dark manipulations show that substate expression is jointly gated by circadian time and ambient light. Together, these results uncover a previously unrecognized, conserved sleep architecture in larval fish, including multiple discrete sleep states with distinct eye-movement kinematics, and establish circadian time and luminosity as key inputs that govern the expression of each state.

## Results

### Identification of sleep periods associated with eye saccades in larval zebrafish

**Fig. 1:**
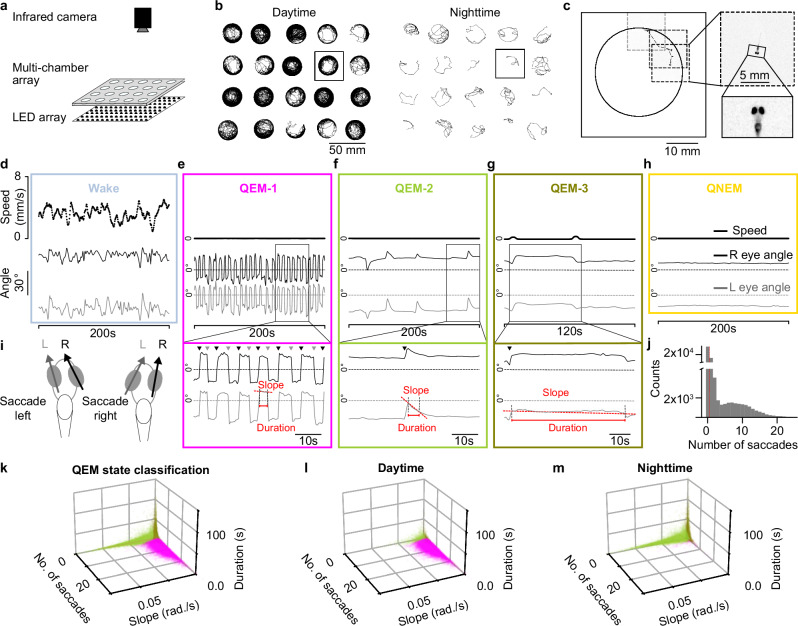
Larval zebrafish sleep comprises distinct substates with different eye movement kinematics. **a** Experimental setup for longitudinal behavioral recordings of larval zebrafish. **b** Example movement trajectories during 1 hour of daytime (left) and nighttime (right). Each larva was housed individually in a 28 × 28 × 1 mm chamber. **c** Real-time tracking at 50 frames per second and 50 µm/pixel resolution enabled detailed eye movement measurements. Dashed boxes indicate the actively tracked region (256 × 256 pixels), which dynamically followed the zebrafish to record behavior at high resolution while minimizing data storage. **d** Speed (top) and eye angles (bottom) during wake for 200 s. **e–h** four distinct sleep periods (sleep defined as by speed <0.5 mm/s for ≥ 1 min). **e** Speed (top) and eye angles (middle) during 200 s of QEM-1; expanded view of eye movements (bottom) with annotated conjugate saccades (gray/black triangles), decay slope (dashed red line), and duration (solid red line). **f–h** as in (**e**), but depicting sleep periods with distinct saccade kinematics (**f**, QEM-2; **g**, QEM-3), and a sleep period with no eye saccades (**h**, QNEM). **i** Conjugate eye saccades were identified as large-amplitude, simultaneous deflections of both eyes in the same direction. **j** Saccade count per 1 min bin during sleep periods (*n* = 105 fish, 24 h recording, 14 h light / 10 h dark). Periods with no saccades (left of dotted red line) were classified as QNEM. **k** For the same 24 h recordings as in (**j**), saccade count, mean decay slope, and mean fixation duration were calculated for every 1 min sleep period and plotted as a single point in a 3D kinematic space (*n* = 105 fish). A 3-class Gaussian Mixture Model (GMM) was used to classify bins into QEM-1, QEM-2, and QEM-3 (pink, green, and olive). **l–m** As in (**k**), but for circadian day (**l**) or circadian night (**m**) only.

To systematically investigate eye movements during spontaneously occurring sleep periods in freely-moving larval zebrafish across the circadian cycle, we developed a new behavioral imaging setup with high spatial resolution (50 µm/px) and high frame rate (50 frames/s) which allowed us to simultaneously record longitudinal behavior of up to 20 freely behaving larval zebrafish for up to three days in static, large, circular chambers (diameter ~28 mm; depth 1 mm; volume ~0.6 ml) (Fig. 1a, b). Briefly, this setup relies on real-time animal tracking of 20 animals in parallel, so that only a small region of interest (ROI) encompassing each animal and its immediate surroundings is saved, allowing us to simultaneously take advantage of a high-resolution camera while incurring a minimal data storage footprint (Fig. 1c, Methods: DASHER). In addition to allowing us to track both locomotion and eye movement in freely moving animals, this setup is equipped with a programmable visible white light emitting diode (LED) array which enables precise control of the lighting conditions during the experiment. Using this setup, we recorded free-swimming behavior of 105 larval zebrafish (5–8 days post fertilization, dpf) over a 24-hour period under light cycled conditions (14-hour light/10-hour dark).

**Fig. 2:**
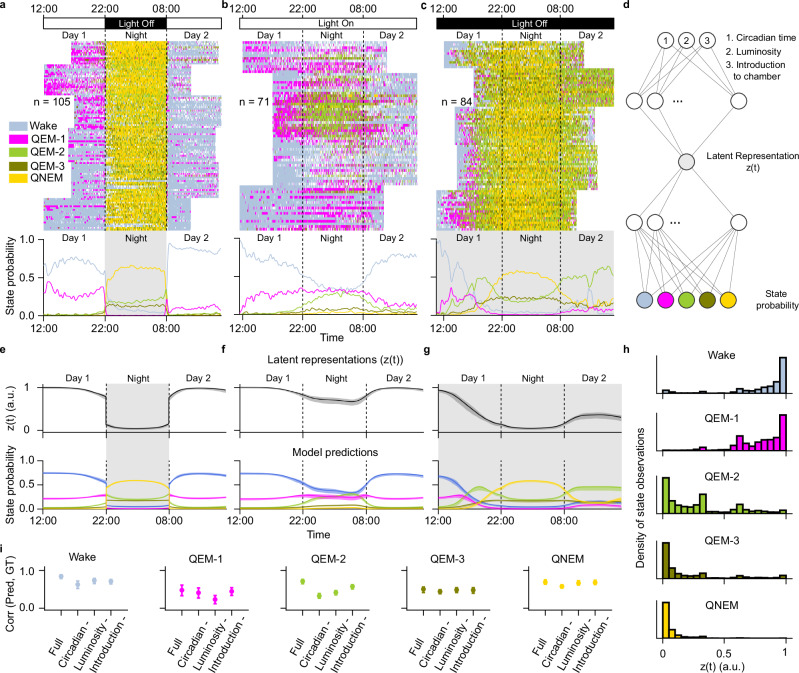
Circadian time and luminosity regulate sleep substates in larval zebrafish. **a** Wake and sleep states over 24 h under light-cycled conditions (14 h light, 10 h dark). Top, raster plot of wake and sleep substates (*n* = 105 fish). Each row represents one larval zebrafish; colors indicate state at each time point. White intervals denote quiescent periods <1 min or tracking errors. Dashed lines mark circadian day-night transitions. Bottom, substate probability computed across fish. Shaded regions indicate lights-off periods. **b**,**c** same analysis for constant-light (**b**, *n* = 71 fish) and constant-dark (**c**, *n* = 84 fish) conditions. **d** State probabilities were modeled as a function of three inputs: circadian time, luminosity, and chamber introduction (indicator function: 1 on day 1, 0 otherwise). The architecture comprises a dense hidden layer (24 units, ReLU), projected to a single latent variable, followed by two dense readout layers (24 units, ReLU; 5-unit softmax output) representing probabilities of Wake, QEM-1, QEM-2, QEM-3, and QNEM, trained on individual fish data from (**a–c**). **e–g** Top, latent representation, z(t), of model inputs under light-cycled (**e**), constant light (**f**) and constant dark (**g**) conditions. Bottom, predicted state probabilities (solid line: mean; shading: 95% CI) for each condition. **h** Distribution of the latent variable z(t) during wake, QEM-1, QEM-2, QEM-3, and QNEM. Each histogram shows the density of observations as a function of z(t) for each substate. All conditions (**a-c**) and fish were pooled for the analysis. **i** Model performance was defined as the Pearson correlation between predicted and observed state probabilities (*n* = 260 fish). Error bars: 95% CIs from 20 repetitions of 5-fold cross-validation (*N* = 100 iterations). Correlations were computed for the full model and following ablation of circadian time, luminosity, or chamber introduction.

During locomotor active periods, zebrafish larvae exhibit frequent swim bouts, with an interbout interval on the order of 1 s^38^. Previous studies have shown that, in addition to locomotor active periods, zebrafish larvae can spontaneously enter sleep periods, which are identified by persistently suppressed locomotor movement for ≥ 1 min and increased arousal thresholds^3,23^. In prior studies using a 14-hour light/10-hour dark cycle, larval zebrafish exhibited sleep periods (lasting ≥1 min) during approximately 5–22% of the circadian day and 50–80% of the circadian night^3,13,23,26,33^. Using this established criterion for sleep (speed <0.5 mm/s for ≥ 1 min, Supplementary Fig. 1), we observed that these sleep periods accounted for 19.6 ± 1.0% of daytime and 83.9 ± 1.5% of nighttime (Fig. 2a; mean ± s.e.; *p*-value = 7.1 ×10^-19^; *n* = 105 fish).

For each sleep period, we then further examined its associated conjugate eye saccade rate and kinematics (Fig. 1e–h, Supplementary Fig. 1). First, we split the data into Quiescent periods with No Eye Movements (QNEM, no saccades in 1 min, Fig. 1h, j; Supplementary Video 1) and Quiescent periods with Eye Movements (QEM, one or more conjugate saccades in 1 min). For the QEM periods, we then fit a three-component Gaussian mixture model to classify each 1 min time bin of QEM based on the number of saccades, average saccade decay slope, and average saccade fixation duration observed during each bin (Supplementary Fig. 1). In this way, each 1 min interval of a QEM period can be further subdivided into three sleep substates with distinct eye movement signatures – QEM-1, QEM-2, or QEM-3 (Fig. 1k).

QEM-1 contained temporally regular conjugate eye saccades (saccade speed = 849.1 ± 158.7 °/s, mean ± s.e., *n* = 11 fish) with high saccade frequency and static, short eye fixation periods (number of saccades = 10.2 ± 4.0 saccades/minute, saccade fixation duration = 4.9 ± 1.8 s, saccade decay slope = 0.32 ± 0.21 °/s, mean ± std, Fig. 1e, Supplementary Video 2, *n* = 105 fish). QEM-2 contained temporally irregular saccades that decayed rapidly (1.6 ± 0.9 saccades/minute, with fixation duration 7.3 ± 5.6 s and decay slope 0.89 ± 0.68 °/s, Fig. 1f, Supplementary Video 3, *n* = 105 fish). QEM-3 also contained temporally irregular, low frequency saccades, but unlike QEM-2 had static and long fixation periods (1.8 ± 2.3 saccades/minute, with fixation duration 9.7 ± 14.9 s and decay slope 0.03 ± 0.07 °/s, Fig. 1g, Supplementary Video 4, *n* = 105 fish).

### Both circadian time and luminosity govern the distribution of sleep substates

Interestingly, we found that the four sleep substates (QEM-1, QEM-2, QEM-3, and QNEM) occupy distinct phases of the circadian cycle. We quantified the sleep substate occupancy for each fish over a 24-hour period (Fig. 2a–c, Supplementary Fig. 2–4). QEM-1 was almost exclusively expressed during circadian day, comprising 81.0 ± 1.9% of total daytime sleep and only 0.3% of total nighttime sleep (Fig. 2a). In contrast, QNEM, QEM-2, and QEM-3 were expressed predominantly during circadian night. QNEM, QEM-2, QEM-3 occupied 64.3 ± 1.1%, 21.0 ± 0.9%, and 14.4 ± 0.7% of nighttime sleep, but only 0.6 ± 0.1%, 13.6 ± 1.6%, and 4.8 ± 0.6% of daytime sleep, respectively.

Furthermore, we observed changes in the relative prevalence of QEM-2 and QNEM over the course of the circadian night (Fig. 2a). The percentage of total time spent in QNEM peaked in the middle of the night, increasing from 38.3 ± 1.9% in the first hour of the night to 63.9 ± 1.7% after 4 h, and then decreasing to 57.8 ± 1.5% in the last hour of the night (p-value start vs middle of night = 4.7 ×10^-17^, *p*-value middle vs end of night = 8.4 ×10^-7^, mean ± s.e., Wilcoxon signed rank test, n = 105 fish). The percentage of total time spent in QEM-2, however, remained stable throughout most of the night (17.0 ± 1.2% in the first hour; 16.4 ± 0.9% in the fifth hour), but increased significantly toward morning, reaching 24.3 ± 1.1% in the final hour of the night (*p*-value start vs end of night = 1.2 × 10^-6^, *p*-value middle vs end of night = 2.9 ×10^-12^, mean ± s.e., Wilcoxon signed rank test, *n* = 101 fish).

To assess whether the temporal organization of the four sleep substates was primarily influenced by environmental luminosity (light vs. dark conditions) or by intrinsic circadian rhythms (time of day), we conducted two additional experiments: 24 h of continuous light (Fig. 2b) and 24 h of continuous darkness (Fig. 2c). Prior to the start of the experiment, animals were reared in normal light cycled conditions. We found that, in both constant light or constant dark experimental conditions, all four states were expressed and exhibited circadian time-dependent dynamics.

Compared to standard light cycled conditions, in constant darkness (Fig. 2c), we observed a large increase in sleep during the day (from 19.6 ± 1.0% to 73.6 ± 1.5% of total daytime) and a smaller increase at night (from 83.2 ± 1.5% to 92.4 ± 0.8% of total nighttime).

The fraction of time spent in QEM-1 was reduced relative to light cycled conditions, but QEM-1 continued to be more prevalent during the circadian day than night (12.3 ± 0.8% of daytime sleep, 0.6% of nighttime sleep, *p*-value = 1.7 ×10^-15^, Wilcoxon signed rank test, *n* = 84 fish). In contrast, the fractions of QNEM and QEM-3 were increased overall during both day and night (Fig. 2c), but continued to be more prevalent during circadian night. QEM-3 accounted for 16.7 ± 0.8% of daytime sleep and 22.8 ± 0.8% of nighttime sleep (*p*-value = 1.9 ×10^-12^, Wilcoxon signed rank test, *n* = 84 fish), while QNEM represented 22.1 ± 1.0% of daytime sleep and 53.4 ± 1.2% of nighttime sleep (p-value = 1.7 ×10^-15^, Wilcoxon signed rank test, *n* = 84 fish). Overall, these results suggest that while complete darkness significantly alters the total amount of sleep, the opposing circadian distributions of QEM-1 with respect to QEM-3 and QNEM is preserved.

Similar to standard light cycled conditions, in constant darkness, the prevalence of QEM-2 increased toward the end of the circadian night. QEM-2 accounted for 18.3 ± 1.0% of total time in the middle of the night and increased to 36.4 ± 1.6% in the hour before the end of the circadian night (*p*-value middle vs end of night = 4.5 ×10^-14^, mean ± s.e., Wilcoxon signed rank test, *n* = 84 fish, Fig. 2c). Unlike in light cycled conditions, we observed increased QEM-2 throughout the circadian day (Fig. 2c). QEM-2 accounted for 48.9 ± 1.2% of daytime sleep. This suggests that both circadian time and luminosity modulate the prevalence of QEM-2.

In constant light conditions (Fig. 2b), the overall amount of daytime sleep remained similar to light cycled conditions (daytime sleep: 26.8 ± 2.5%, *n* = 71 fish). QEM-1 was enriched during circadian day (72.5 ± 2.7% of total daytime sleep, mean ± s.e., *n* = 71 fish). At night, constant light decreased the amount of overall sleep dramatically (nighttime sleep: 48.3 ± 3.7%; *n* = 71 fish), as reported previously^39^. However, constant light also altered the composition of sleep at night, with QEM-1 occupying 56.9 ± 3.6% of total nighttime sleep (mean ± s.e., *n* = 70 fish). QEM-2 increased to 31.3 ± 2.7%, while QEM-3 and QNEM decreased (9.1 ± 0.9% and 2.7 ± 0.5% of nighttime sleep, respectively). Thus, ambient light at night promoted QEM-1 while suppressing other states, in particular QEM-3 and QNEM.

### Daytime sleep decreases from day 1 to day 2, but QEM-1 and QNEM states continue to occupy distinct phases of the circadian cycle

In addition to luminosity and circadian rhythm, we observed that the overall probability of daytime sleep is higher on day 1 compared with day 2 (Fig. 2a–c). To control for the possibility that stress from animal handling increased daytime sleep on day 1, we conducted an additional set of experiments in which we loaded the fish into the behavioral chamber at 4 dpf for 16-17 h of acclimatization (day 0) prior to the beginning of the experiment. However, after acclimatization on day 0, the percentage of daytime sleep still decreased between day 1 and 2 (Supplementary Fig. 2a–c; day 1, 5 dpf: 30.8 ± 4.0%; day 2, 6 dpf: 21.7 ± 3.8%; mean ± s.e.; *p*-value = 0.09; n = 27 fish). This suggests that animal loading and handling does not entirely account for the higher level of daytime sleep on day 1 compared to day 2. One possibility to consider in future studies is that 4 dpf animals are less spontaneously exploratory than older animals, and thus, the increased sleep on day 1 may be coupled to self-initiated spatial exploration of novel environments, which begins at 5 dpf.

Though the total amount of daytime sleep decreases on day 2, the circadian distribution of QEM and QNEM states continues to hold across days. Daytime sleep was still dominated by QEM-1 (Supplementary Fig. 2c; out of total daytime sleep: 83.9 ± 3.0% on day 1 and 76.6 ± 3.8% on day 2), and nighttime sleep was mostly occupied by QNEM (Supplementary Fig. 2c; out of total nighttime sleep: 70.8 ± 1.7% on night 1 and 68.7 ± 2.6% on night 2). QEM-2 increased towards the end of circadian night on both nights (Supplementary Fig. 2a). Thus, pre-loading the animal at 4 dpf does not alter the circadian distribution of QEM and QNEM states.

We next considered the possibility that older animals ( > 5 dpf) may require increased water flow for oxygen delivery, as opposed to passive gas exchange through our gas-permeable chamber. To test this, we designed an additional behavioral setup (diameter ~28 mm; depth 1 mm; volume ~0.6 ml) that enables continuous flow of oxygenated water at a low flow rate ( ~ 0.5 mL/min) throughout the entire 48-hour experimental period (Methods: Water flow experiments). Using this behavioral setup (Supplementary Fig. 3a–c, Methods), we find a similar decrease in daytime sleep from day 1 to day 2. Sleep periods comprised 38.9 ± 5.1%, 20.6 ± 3.6%, and 21.9 ± 4.1% of days 1–3, respectively (*p*-value day 1 vs day 2 = 3.1 ×10^-3^, *p*-value day 2 vs day 3 = 0.823, Wilcoxon signed rank test, mean ± s.e., *n* = 23 fish). The lack of significant change in daytime sleep between day 2 and 3 suggests that the probability of daytime sleep stabilizes after day 1.

Across all three days (Supplementary Fig. 3c), QEM-1 comprised the largest fraction of daytime sleep, although this fraction decreased over time (82.1 ± 4.8%, 78.6 ± 4.2%, 51.5 ± 7.5% on day 1, 2, and 3, respectively, mean ± s.e., *n* = 23 fish). Conversely, QEM-2 accounted for an increasing fraction of daytime sleep across days (9.1 ± 2.4%, 14.8 ± 3.2%, 28.5 ± 4.5% on day 1, 2, and 3, respectively). QNEM and QEM-3 exhibited nearly identical contributions to total daytime sleep across all observed days (QNEM: 2.9 ± 2.2%, 0.9 ± 0.3%, and 5.0 ± 1.5% on days 1, 2, and 3, respectively; QEM-3: 5.8 ± 1.8%, 5.6 ± 1.2%, and 15.0 ± 3.8% on days 1, 2, and 3, respectively). The relative increase of QEM-2 during circadian day may be a consequence of changes in the age or internal state of the animal (e.g., the animals were not fed during the extended recording).

### An ANN-based logistic regression model can predict sleep substates with a single latent

To quantify the relative dependence of each QEM and QNEM sleep substates on environmental (luminosity and time since introduction to the environment) vs. internal (circadian time) factors, we built an artificial neural network-based logistic regression model which transforms these three inputs to a prediction of state probability at each point in time. We modeled this transformation using a 4-layer neural network consisting of an input layer mapping to a scalar latent variable, followed by a 2-layer readout. All layers were dense, with non-linear activations (Fig. 2d, Methods: ANN logistic regression model). The predicted sleep substate probabilities for all experimental conditions can largely recapitulate the ground truth sleep substate probabilities (Pearson correlation for QEM-1: 0.48 ± 0.07, QEM-2: 0.72 ± 0.03, QEM-3: 0.50 ± 0.04, QNEM: 0.69 ± 0.03, mean ± s.e.; Fig. 2e–i). Interestingly, distinct substates map to different regions of the model’s 1-D latent variable (Fig. 2h), suggesting that a latent axis may underlie the relative expression of each sleep substate over time.

To directly measure the relative contribution of each model input to sleep substate probability, we ablated one input variable at a time and measured the change in performance between the ablated model and the full model (Fig. 2i; Methods: ANN logistic regression model). Our results indicate that all four sleep substates depend significantly on circadian time (QEM-1: Δr = -0.07, *p* = 8.39e-9; QEM-2: Δr = 0.39, *p* = 1.95e-18; QEM-3: Δr = 0.06, *p* = 3.35e-18; QNEM: Δr = 0.11, *p* = 1.94e-18), luminosity (QEM-1: Δr = 0.25, *p* = 1.95e-18; QEM-2: Δr = 0.30, *p* = 1.95e-18; QEM-3: Δr = 0.02, *p* = 3.35e-13; QNEM: Δr = 0.03, *p* = 1.94e-18), and introduction to the chamber (QEM-1: Δr = 0.03, *p* = 4.17e-7; QEM-2: Δr = 0.14, *p* = 1.95e-18; QEM-3: Δr = 0.02, *p* = 3.25e-13; QNEM: Δr = 0.01, *p* = 1.77e-17; Wilcoxon signed rank test). Although all sleep substates depend significantly on all three model inputs, the relative impact of each variable varied by sleep state. Notably, the novel daytime sleep state, QEM-1, depended more strongly on luminosity while the nighttime sleep state QNEM depended more strongly on circadian time.

We note that while the model captures the dynamics of sleep substates over 24 h, there are additional changes in the distribution of sleep substates in constant light conditions over 48 h. In constant light over 48 hours (Supplementary Fig. 3d–f), we observed a significant increase in QEM-2 and QNEM during the second night. QEM-2 accounted for 22.2 ± 4.2% of total sleep on night 1 and 40.0 ± 4.1% on night 2 (mean ± s.e., *n* = 12 fish). QNEM accounted for 2.1 ± 1.1% of total sleep on night 1 and 23.7 ± 4.3% on night 2 (Supplementary Fig. 3f). As the animals were reared in normal light cycled conditions prior to the start of the experiment, the sudden decrease in QNEM, QEM-2, and QEM-3 on night 1 may affect the distribution of these states on subsequent nights. Extending the model to incorporate the history of sleep substates and sensory experiences (e.g., over previous days and nights) may be interesting to explore in future studies.

### All sleep substates are expressed by closely related *Danio* species and zebrafish strains

**Fig. 3:**
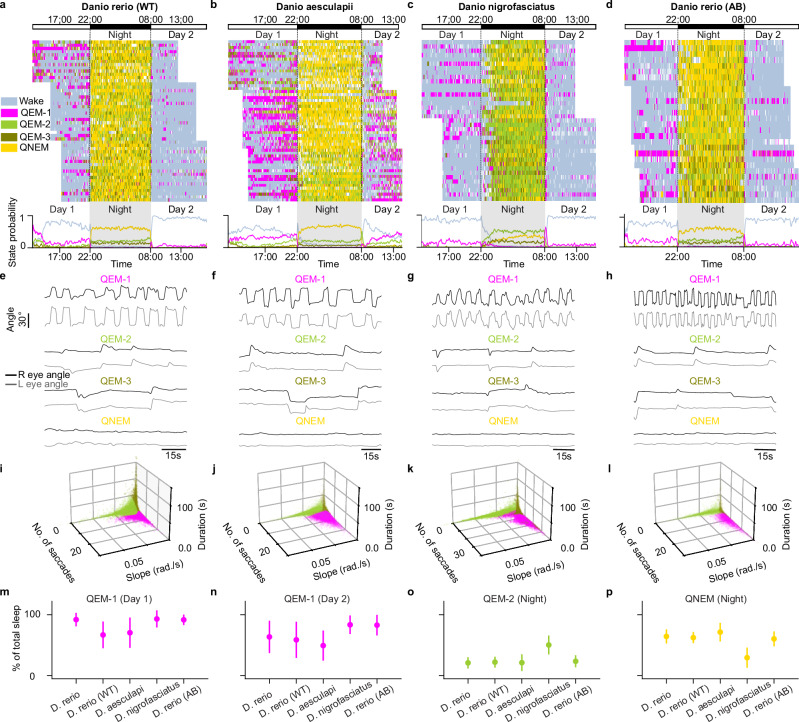
Sleep substates persist in closely related Danio species and zebrafish strains. **a–d** Behavioral recordings over 24 hours under light-cycled condition (14 h light, 10 h dark) for wild-caught *Danio rerio* (**a**, *n* = 58 fish), *Danio aesculapii* (**b**, *n* = 57 fish), *Danio nigrofasciatus* (**c**, *n* = 37 fish), and AB strain *Danio rerio* (**d**, *n* = 28 fish). Top, raster plot of classified wake and sleep substates. Each row represents an individual animal, with colors indicating wake and sleep substate classification at each time point. White intervals denote quiescent periods shorter than 1 min and instances of fish or eye tracking errors. Dashed black lines mark circadian transitions between day and night. Bottom, probability of each sleep substate, computed across all animals. Shaded regions indicate when the lights were off at night. **e**–**h** Eye angle kinematics for all QEM states in WT *Danio rerio* (**e**), *Danio aesculapii* (**f**), *Danio nigrofasciatus* (**g**), and AB strain *Danio rerio* (**h**)**. i–l** Number of saccades, mean saccade decay slope, and mean saccade duration for each 1 min sleep period for WT *Danio rerio* (**i**, *n* = 58 fish), *Danio aesculapii* (**j**, *n* = 57 fish), *Danio nigrofasciatus* (**k**, *n* = 37 fish), and AB strain *Danio rerio* (**l**, *n* = 28 fish). Colors indicate state classification based on a three-component Gaussian Mixture Model (GMM) that was fit on our lab strain *Danio rerio* (mitfa-/- TLAB, “*D. rerio*”) (Fig. 1k) and then applied to each species or zebrafish strain. **m–p** Time spent in each sleep substate (**m**, QEM-1 on day 1; **n**, QEM-1 on day 2; **o**, QEM-2 at night; **p**, QNEM at night) as a percentage of total sleep for WT *Danio rerio* (*n* = 58 fish), *Danio aesculapii* (*n* = 57 fish), *Danio nigrofasciatus* (*n* = 37 fish), AB strain *Danio rerio* (*n* = 28 fish), and TLAB *Danio rerio* (*n* = 105 fish). Data are presented as mean ± s.d.

To investigate whether the sleep substates observed in larval zebrafish (*Danio rerio)* were specific to this species or to our lab strain (mitfa -/- TLAB), we conducted additional behavioral experiments in closely related *Danio* species and distinct zebrafish strains (Fig. 3). These included AB strain zebrafish (Erwin Van Wijk’s lab at Radboud University), wild-caught pet-store *Danio rerio* (WT), *Danio nigrofasciatus*, and *Danio aesculapii*. We used the same criteria to define sleep periods (speed <0.5 mm/s for ≥ 1 min) across all species. We extracted saccade properties (number of saccades, mean saccade decay slope, saccade fixation duration) as above, and utilized the same state classification boundaries from our light-cycled *Danio rerio* data in Fig. 1. We found that all four sleep substates were clearly present in each of the additional strains and species tested (Fig. 3a–h).

Strikingly, the circadian distribution of QEM-1 (enriched during the day) and QEM-2, QEM-3, and QNEM (all enriched during the night) was generally preserved across strains and species (Fig. 3m–p). Furthermore, QEM-2 increased progressively throughout the circadian night in the *D. rerio* WT (Fig. 3a) and *D. rerio* AB strains (Fig. 3d). Using one hour analysis windows at the start, middle and end of circadian night, we find that in the *D. rerio* WT strain, QEM-2 accounted for 18.0 ± 1.3% of total time at the start of the night, 19.4 ± 1.4% in the middle of the night and 27.2 ± 2.0% at the end (*p*-value start vs end = 1.9 × 10^-5^, *p*-value middle vs end = 9.9 ×10^-5^, *n* = 50 fish). Similarly, in the *D. rerio* AB strain, QEM-2 accounted for 16.6 ± 2.3% of total time at the start of the night, 20.5 ± 2.1% in the middle of the night and 33.1 ± 2.8% at the end (p-value start vs end = 1.2 ×10^-4^, *p*-value middle vs end = 3.1 ×10^-4^, *n* = 28 fish).

Finally, we did note some subtle, but potentially important, differences between species. *Danio aesculapii* (Fig. 3b) was unique in exhibiting similar levels of daytime sleep across days, with 38.0 ± 2.9% on day 1 and 32.4 ± 2.6% on day 2 (*p* = 0.159, Wilcoxon signed rank test, *n* = 55 fish), suggesting these animals may be less sensitive to initial introduction into a novel environment compared to other species and strains tested. Additionally, unlike all other species and strains, the predominant nighttime sleep in *Danio nigrofasciatus* was not QNEM but QEM-2 (Fig. 3c, o, p). QNEM represented only 29.5 ± 2.7% of total nighttime sleep, while QEM-2 accounted for 50.3 ± 2.5% (*p* = 2.9 ×10^-4^, Wilcoxon signed rank test, *n* = 37 fish).

Performing this analysis using species-specific state classification boundaries did not change our conclusions (Supplementary Fig. 5). Overall, these data suggest a robust conservation of the circadian organization of sleep substates, along with intriguing species-specific modulation of overall substate occupancy.

### Circadian segregation of sleep substates persists, independent of chamber size or water temperature

To determine whether specific water temperature or chamber size influenced the presence or circadian distribution of the four sleep substates, we repeated the 24-hour experiments under different combinations of temperature and water depth (Supplementary Fig. 6). We found that all four substates were present in all conditions, with similar circadian distributions: QEM-1 was enriched during daytime sleep, while QEM-2, QEM-3, and QNEM were enriched during nighttime sleep.

At 1 mm water depth and 24 °C (total volume: ~0.6 ml; Supplementary Fig. 6a, c; *n* = 49 fish), we observed an overall sleep of 8.1 ± 1.5% during circadian daytime and 84.2 ± 1.6% during circadian nighttime. QEM-1, QEM-2, QEM-3, and QNEM accounted for 73.3 ± 2.4%, 15.3 ± 1.6%, 9.5 ± 1.3%, and 1.8 ± 0.6% of daytime sleep, respectively, as well as 0.3 ± 0.0%, 16.7 ± 1.0%, 20.0 ± 1.4%, and 63.0 ± 1.7% of nighttime sleep, respectively.

At 1 mm water depth and 28 °C (total volume: ~0.6 ml; Supplementary Fig. 6b, c; *n* = 42 fish), we observed an overall sleep, as traditionally defined, of 5.9 ± 1.1% during circadian daytime and 79.8 ± 1.6% during circadian nighttime. QEM-1, QEM-2, QEM-3, and QNEM accounted for 84.6 ± 1.9%, 9.3 ± 1.4%, 5.5 ± 0.8%, and 0.7 ± 0.3% of daytime sleep, respectively, as well as 0.9 ± 0.3%, 18.2 ± 1.4%, 26.8 ± 1.2%, and 54.7 ± 1.6% of nighttime sleep, respectively.

At a water depth of 10 mm and a temperature of 24 °C (total volume: ~6 ml; Supplementary Fig. 6d, f; *n* = 25 fish), we observed an overall sleep of 9.9 ± 2.5% during circadian daytime and 59.2 ± 4.4% during circadian nighttime. QEM-1, QEM-2, QEM-3, and QNEM accounted for 60.6 ± 6.3%, 20.5 ± 4.5%, 12.8 ± 2.9%, and 6.2 ± 1.5% of daytime sleep, respectively, as well as 0.3 ± 0.1%, 9.9 ± 0.8%, 39.0 ± 3.1%, and 50.8 ± 3.2% of nighttime sleep, respectively.

At 28 °C and 10 mm water depth (total volume: ~6 ml; Supplementary Fig. 6e, f; *n* = 41 fish), we observed an overall sleep of 4.1 ± 1.0% during circadian daytime and 63.0 ± 3.3% during circadian nighttime. QEM-1, QEM-2, QEM-3, and QNEM accounted for 75.4 ± 3.8%, 8.2 ± 2.1%, 14.5 ± 2.6%, and 1.9 ± 1.1% of daytime sleep, respectively, as well as 0.7 ± 0.2%, 18.1 ± 1.2%, 27.8 ± 1.6%, and 53.4 ± 2.4% of nighttime sleep, respectively.

### QEM-1 is associated with an increased arousal threshold

**Fig. 4:**
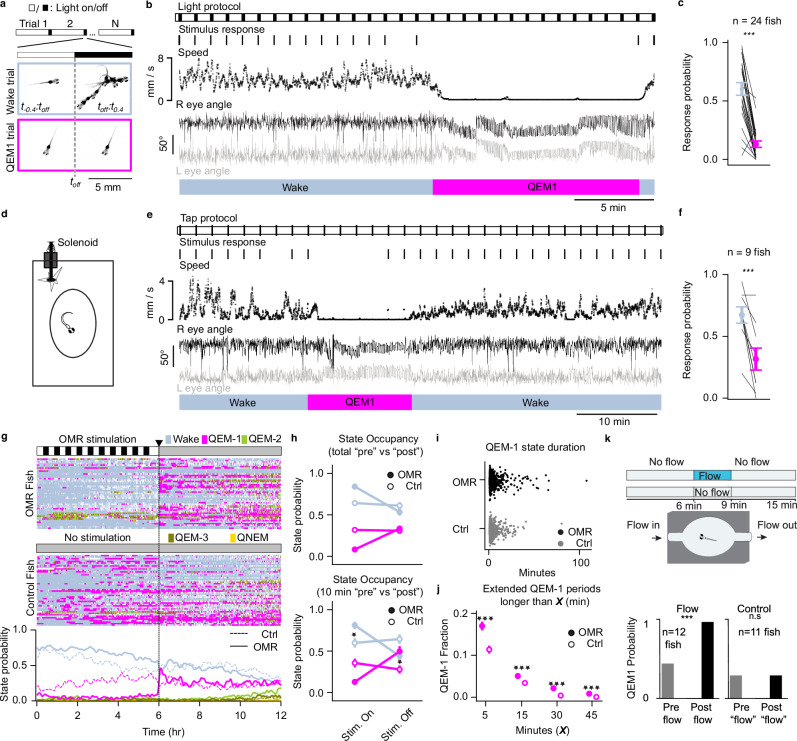
QEM-1 is a low-arousal state. **a** Dark flash protocol: 1 s stimulus, 60 s inter-trial interval. Bottom, example trials during wake (top) and QEM-1 (bottom), 10 overlaid images spanning 400 ms. **b** 30 min excerpt showing speed and eye angles during a dark flash experiment. **c** Startle probability by state: significantly higher during wake (mean ± s.e., *p* = 8.3 × 10^-7^, *n* = 24 fish, Wilcoxon signed-rank test). **d** Mechano-acoustic tapping setup: solenoid struck chamber plate every 2 min. **e** 60 min excerpt from a tapping experiment. **f** Startle probability by state: significantly higher during wake (mean ± s.e., *p* = 0.01, *n* = 9 fish, Wilcoxon signed-rank test). **g** Stimulated fish (*n* = 47) were exposed to moving gratings for 6 h then uniform illumination for 6 h to test QEM-1 deprivation effects; controls (*n* = 48) received uniform illumination for 12 h. Raster plots show per-fish state occupancy (top, middle); bottom shows group averages for stimulated (solid) and control (dashed) fish. **h** Mean wake and QEM-1 probability during stimulus (Stim. On) and test (Stim. Off) periods, over the full 6 h or 10 min before and after stimulus offset. In the first 10 min post-stimulation, QEM-1 probability was significantly higher in deprived than control fish (mean ± s.e., *p* = 0.026, rank-sum test). **i** QEM-1 bout duration distribution for deprived (black) and control (gray) fish. **j** QEM-1 duration comparison post-deprivation (deprived *n* = 47; control n = 48). X-axis: duration thresholds; y-axis: fraction of total QEM-1 periods, for control (unfilled circle) and deprived (filled circle): 5 min (*p* = 6.25 × 10^-33^); 15 min (*p* = 1.76 × 10^-06^); 30 min (*p* = 4.08 × 10^-34^); 45 min (*p* = 3.02 × 10^-34^); all mean ± s.d., Wilcoxon rank-sum test. **k** Stimulated fish received 3 min water flow after 6 min baseline; controls received no flow. QEM-1 probability was significantly higher post-flow in stimulated fish (*p* = 0.0004, *n* = 12, Binomial test) but not controls (mean probability, *p* = 1.0, *n* = 11, Binomial test).

QEM-1 is associated with a unique eye saccade signature and is almost exclusively expressed during circadian daytime. To determine whether QEM-1 is indeed a low arousal sleep state, we assessed QEM-1 arousal thresholds using two distinct sensory modalities (Fig. 4a–f). First, we presented fish with a series of “dark flash” visual stimuli, which is known to elicit large amplitude startle responses in larval zebrafish^40^. Dark flashes were presented once per minute with a stimulus duration of 1 s, and startle response probability was quantified as the fraction of trials where a startle movement occurred during this 1 s window (Fig. 4a, Methods: Dark flash arousal assay). In this window, we found high startle response probability during wake (0.60 ± 0.05, mean ± s.e.) and significantly suppressed startle response probability during QEM-1 (0.13 ± 0.03, mean ± s.e., *p*-value = 8.3 ×10^-7^, *n* = 24 fish, Wilcoxon signed rank test, Fig. 4b, c), indicating an increase in arousal threshold during QEM-1. By definition, movement levels were at or near zero during the QEM-1 state. Therefore, we performed a control analysis to ensure that the difference in startle response probability between wake and QEM-1 could not be statistically explained by changes in overall movement levels between the two states (Supplementary Fig. 7a, 7c, d). After controlling for changes in gross motor movement between the two states, response probability remained reduced, confirming that arousal threshold is increased during QEM-1. Finally, to ensure that our results were not due to fish adapting to the regularity of the dark flash stimulus, we repeated the experiments on a new batch of fish but randomized the timing of stimulus presentations. In the randomized setting, dark flash responses remained significantly suppressed during QEM-1 (wake: 0.58 ± 0.07, QEM-1: 0.14 ± 0.04, p-value = 4.9 ×10^-4^, *n* = 12 fish, Wilcoxon signed rank test) (Supplementary Fig. 7g).

Second, we tested whether sensory modalities other than vision were also affected by QEM-1. To do this, we performed a mechano-acoustic tapping assay, which, like dark flashes, elicits startle responses in alert fish^41^ (Fig. 4d, Methods: Mechano-acoustic tap arousal assay), and has been used to quantify arousal levels across sleep and wake^33,37^. Mechano-acoustic taps were delivered once every 2 min, and the response window was defined as the 1 s period following stimulus onset. Again, the startle response probability to mechano-acoustic taps was significantly reduced during the QEM-1 state (wake: 0.67 ± 0.07, QEM-1: 0.32 ± 0.09, mean ± s.e., *p* = 0.01, *n* = 9 fish, Wilcoxon signed rank test; Fig. 4e, f) and this effect could not be explained by the decreased overall movement levels during locomotor quiescence (Supplementary Fig. 7b, 7e, f).

Because this stimulus was not visual, we could also perform this arousal assay in the dark to probe arousal levels during the nighttime sleep substates as well. Both QEM and QNEM exhibited significant increase in arousal thresholds relative to wake (Supplementary Fig. 7i), fulfilling a key criterion of sleep^3^. Interestingly, QEM-1 had the lowest startle response probability (indicating the highest arousal threshold), followed, in order, by QEM-2, QEM-3, and QNEM (wake: 0.71 ± 0.09, QEM-1: 0.21 ± 0.13, QEM-2: 0.42 ± 0.02, QEM-3: 0.62 ± 0.06, QNEM: 0.68 ± 0.05, *n* = 5 fish, Supplementary Fig. 7i). Similar to dark flashes, in the randomized setting, startle responses to mechano-acoustic taps remained significantly suppressed during QEM-1 across all tap strengths (Supplementary Fig. 7h).

### QEM-1 is expressed in blind fish, suggesting that QEM-1 associated conjugate eye saccades do not require visual feedback

The presence of QEM-1 during constant darkness suggests that visual feedback is not necessary to maintain QEM-1. However, it remains possible that these QEM-1 eye movements in the dark depend on past visual experience. To test this, we performed 24-hour experiments with *lakritz-/-* mutant fish which lack retinal ganglion cells^42^ and are therefore congenitally blind. We identified *lakritz-/-* mutants by their characteristic hyperpigmentation (Supplementary Fig. 8a) and confirmed the blindness of the hyperpigmented animals by their lack of responsiveness to visual stimuli (Supplementary Fig. 8b, c). Consistent with this blindness phenotype, these hyperpigmented *lakritz-/-* mutants displayed significantly lower optomotor response (OMR) in the stimulus direction (-0.12 ± 0.01 mm/s, mean ± s.e., *n* = 11 fish) than that of *lakritz* + */+; mitfa-/-* controls (0.35 ± 0.10 mm/s, mean ± s.e., p-value = 1.8 ×10^-3^, *n* = 5 fish, Wilcoxon rank sum test). Remarkably, *lakritz-/-* mutants clearly expressed QEM-1 states (Supplementary Fig. 8d, e). These results indicate that temporally regular conjugate eye saccades are intrinsically part of the QEM-1 state irrespective of visual input, reminiscent of observations in congenitally blind humans^43,44^.

### QEM-1 is associated with partial loss of postural control

To investigate whether partial loss of postural control^36,45,46^ is associated with QEM-1, we measured the roll and pitch of animals during QEM-1 and wake states using calcium imaging of whole brain activity (Supplementary Fig. 9, Methods: High-resolution offline registration of fluorescent brain volumes). While no significant difference in pitch was observed between wake and QEM-1 (*p* > 0.05, *n* = 11 fish, Wilcoxon signed-rank test), roll exhibited clear state-dependent changes (Supplementary Fig. 9a-c). During wakefulness, roll was symmetrically distributed around zero (Supplementary Fig. 9b), reflecting active muscle contraction to maintain upright posture^47^. In contrast, during QEM-1, roll was significantly skewed (either positively or negatively) throughout the state (mean absolute roll during wake: 3.31 ± 0.29° vs. QEM-1: 5.29 ± 0.77°, mean ± s.e., p-value = 0.01, n = 11 fish, Wilcoxon signed-rank test, Supplementary Fig. 9c). These findings indicate that QEM-1 is associated with a partial loss of postural control.

To preclude the possibility that eye movements during QEM-1 are simply compensating for the roll of the fish, we computed the average correlation of left and right eye movements with roll for each QEM-1 period and averaged across all the QEM-1 periods for each animal (Supplementary Fig. 9d). We found no significant correlation between eye movements and roll of the larval zebrafish during QEM-1 (correlation: 0.03 ± 0.04, mean ± s.e., *n* = 10 fish, Supplementary Fig. 9e) suggesting that loss of postural control is independent of eye movements during QEM-1.

### Extended deprivation of daytime sleep leads to rebound in QEM-1 state

In addition to loss of postural control, a hallmark of sleep is homeostatic regulation^48,49^. This prompted us to explore whether QEM-1 is also subject to homeostatic regulation. To do this, we deprived fish of daytime sleep (predominantly QEM-1) for several hours and evaluated the amount of QEM-1 following deprivation relative to sibling control fish which were not deprived. We used moving visual gratings as our deprivation stimulus as they are known to elicit robust directional movement via the OMR in larval zebrafish^50,51^ and have been used previously to induce prolonged periods of wakefulness^26^.

Specifically, we presented moving gratings during the daytime for 6 h, with the direction of motion changing every 15 s to prevent adaptation to the stimulus (Fig. 4g). We observed a significant increase in wake state probability relative to control siblings during the deprivation period (deprived: 0.84 ± 0.03, control: 0.64 ± 0.04, mean ± s.e., p-value = 2.8 ×10^-5^, OMR *n* = 47 fish, control *n* = 48 fish, Rank sum test), indicating that our protocol partially decreased the total amount of daytime sleep (Fig. 4g, h). In the first 10 min following deprivation, we observed significantly higher QEM-1 state probability relative to control fish (Fig. 4h, deprived: 0.49 ± 0.06, control: 0.28 ± 0.05, mean ± s.e., *p*-value = 0.026, OMR *n* = 47 fish, control *n* = 48 fish, Rank sum test). After this initial 10-minute period, QEM-1 probability returned to approximately the same level as control fish. Despite this relatively short rebound in QEM-1 probability, we observed a robust, prolonged increase in the duration of QEM-1 periods relative to controls during the remaining 6 daytime hours following deprivation (Fig. 4i, j, deprived: 4.01 ± 0.13 min, control: 3.02 ± 0.22 min, p-value = 5.7 ×10^-3^, mean ± s.e., OMR *n* = 47 fish, control *n* = 48 fish, Rank sum test). This suggests that while the overall amount of QEM-1 increased only modestly following deprivation, when deprived fish entered QEM-1 they did so for longer, more consolidated periods than control fish.

One limitation of using a moving grating deprivation protocol is that the overall movement vigor, conditioned on a fish being awake and moving, could potentially be higher relative to control movement levels. Thus, it is possible that the rebound in QEM-1 probability and duration is a response to general fatigue, rather than selective deprivation of the QEM-1 state. To address this, we measured the average displacement and peak bout speed during wakefulness for both deprived and control larvae and repeated the analysis on the subset of fish with matching movement statistics between groups (Supplementary Fig. 10a, b). In these matched animals, we again observed a robust increase in the duration of QEM-1 periods following deprivation (Supplementary Fig. 10c, d). These findings suggest that the rebound in QEM-1 probability and state duration is likely not simply motor fatigue.

A recent study in zebrafish also demonstrated that inducing an increase in global brain-wide activity using pharmacology leads to an immediate and robust rebound in sleep^26^. Similarly, we found that using water flow as a naturalistic perturbative stimulus triggered a sudden and persistent increase in many neurons’ activity throughout the brain which was followed by an increase in QEM-1 probability (Supplementary Fig. 11). During 1 min of constant flow (3.7 ± 0.58 ml/min, mean ± s.d., *n* = 10 fish), 62% of neurons increased their activity relative to pre-flow baseline activity (Supplementary Fig. 11). Behaviorally, water flow significantly increased the probability of observing QEM-1 during a six-minute window following the flow stimulus (post-flow probability: 0.92 vs pre-flow QEM-1 probability: 0.42, p-value = 4.0 ×10^-4^, *n* = 12 fish, Binomial Test, Fig. 4k). Thus, on short timescales, naturally induced increases in brain activity are correlated with subsequent upregulation of the QEM-1 state.

### Identification of neurons encoding behavioral and state variables in QEM-1

To investigate the neural dynamics of QEM-1, we performed brain-wide calcium imaging in freely swimming animals expressing pan-neuronal H2B-GCaMP6s or H2B-GCaMP8s (5–8 dpf) while continuously recording both locomotion and eye movements^52^. Putative single neuron activity traces were extracted using Non-negative Matrix Factorization^31^ (Methods: NMF). By combining neural and behavioral imaging, we were able to identify three key neural features of QEM-1. First, QEM-1 is associated with a broad suppression of neural activity; second, distinct populations of neurons encoded the behavioral features of QEM-1 (e.g., eye saccades) vs. the QEM-1 state itself; and third, global brain dynamics evolved in an organized way across time during the QEM-1 state.

**Fig. 5:**
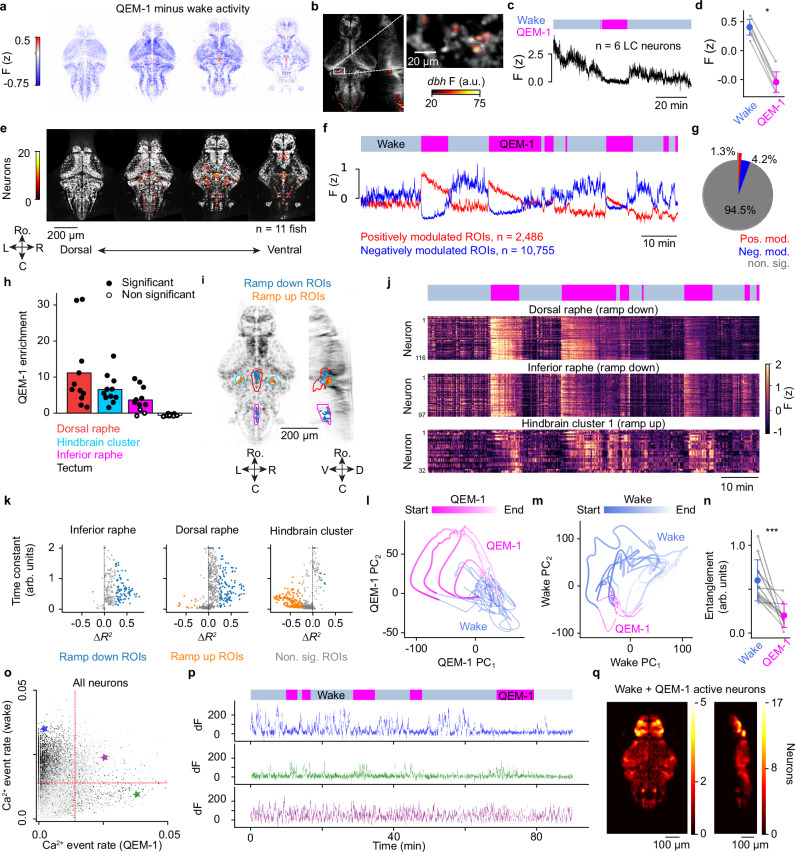
Brain-wide analysis reveals QEM-1 state encoding neural populations. **a** Neural activity during QEM-1 minus activity during wake, averaged across neurons in each bin (*n* = 11 fish). Split into 5 dorsoventral quantiles; the four most dorsal are shown. **b** Reference brain with colormap indicating dbh in-situ labeling intensity. Inset highlights the left locus coeruleus (LC). **c** Mean raw fluorescence across the six *dbh*-positive LC cells shown in (**b**). **d** Mean LC calcium activity during wake and QEM-1 (mean ± s.e., *p* = 0.03, *n* = 6 fish, Wilcoxon signed-rank test). **e** Heatmap intensity indicates the number of positively modulated QEM-1 neurons per location; same dorsoventral sections as in (**a**). **f** Mean activity of positively (red) and negatively (blue) modulated neurons in one example fish. **g** Fraction of neurons that were unmodulated (gray), negatively modulated (blue), or positively modulated (red) during QEM-1 across fish. **h** Fold-enrichment of positively modulated neurons per brain region relative to random anatomical sampling (bar plot). Each overlaid dot represents one fish (one-sided permutation test, *p* < 0.05 fill circles, *p* ≥ 0.05 unfilled circles). **i** Anatomical distribution of positively modulated QEM-1 neurons. **j** Activity rasters for one example fish. **k** Exponential model fits for positively modulated neurons (*n* = 11 fish). Each dot is one neuron, categorized as ramp-up (orange), ramp-down (blue), or non-ramping (gray). ΔR^2^ = R^2^ of exponential decay minus R^2^ of “1 minus exponential” ramp-up model. **l** Whole-brain PCA during QEM-1. Timepoints projected onto the top two PCs, colored by state and time within each period. **m** As in (**l**), with PCA performed on wake periods. **n** Mean trajectory entanglement per fish (mean ± s.d., *p* = 0.001, *n* = 11 fish, Two-sided Wilcoxon signed-rank test). **o** Neuronal activity event rates during wake and QEM-1. Each dot represents one neuron. Red lines: threshold (0.014 events/s). **p** Example neurons active only during wake (top), only during QEM-1 (middle), or both (bottom). **q** Anatomical distribution of neurons active during both states (*n* = 10 fish), using the thresholds defined in (**o**). Intensities represent smoothed densities in binned brain space.

To determine how overall brain activity changed between QEM-1 and wake, we measured mean neural activity of each neuron during each state. During QEM-1, neural activity was broadly suppressed across the brain (Fig. 5a, Supplementary Video 5). Across fish (*n* = 11 fish), 73.7 ± 4.2% (mean ± s.d.) of all neurons were negatively correlated with the QEM-1 state. Using post-hoc in-situ labeling of noradrenaline-expressing cells^53^, we found that the noradrenergic locus coeruleus was strongly suppressed during QEM-1 (Fig. 5b–d), supporting our behavioral evidence that QEM-1 is a low arousal sleep state^25,54^.

Despite broad suppression of neural activity, a subset of brain regions was significantly more active during QEM-1 than during wake (Fig. 5e). We hypothesized that neurons in these brain regions could encode either the characteristic motor patterns of QEM-1 (e.g., eye saccades), or the QEM-1 state itself. To distinguish between these two possibilities, the activity of each neuron was regressed against both behavioral (eye movement, swimming speed, etc.) and QEM-1 state variables using multivariate linear regression (Methods: Multivariate Linear Regression Analysis). To determine which aspects of behavior and/or internal state were encoded by single neurons, we compared the full model performance (R^2^) to a set of additional partial models in which specific subsets of variables were excluded by temporal shuffling (Methods: Multivariate Linear Regression Analysis). A regression variable contributed significantly to a neuron’s activity if the full model significantly outperformed the partial model with that variable removed.

As validation of our approach, we found that neurons encoding QEM-1 associated eye movements were primarily located in the abducens nucleus, consistent with prior work^55^ (Supplementary Fig. 12a, b). Interestingly, unlike previous studies in immobilized animals^56^, we found a clear anatomical segregation of saccade encoding neurons from neurons encoding left and right turn bias (Supplementary Fig. 12b). In waking states, locomotor neurons were active, while saccade neurons were largely suppressed (Supplementary Fig. 12c, d). This is consistent with our behavioral observation that large amplitude, stable eye saccade events are mostly decoupled from swimming movements (Supplementary Fig. 12c, d).

In addition to neurons encoding behavioral features of QEM-1, we identified 991 ± 720 (mean ± s.d., *n* = 11 fish) neurons per fish that uniquely and positively encoded the QEM-1 state itself (Fig. 5e–g). These putative QEM-1 neurons were concentrated primarily in the ventral hindbrain and made up 1.3% of all recorded neurons, on average (Fig. 5g). Specifically, these QEM-1-encoding cells were significantly enriched in the dorsal and inferior raphe, as well as in an adjacent lateral hindbrain cluster (fold enrichment in dorsal raphe: 12.2 ± 10.6, inferior raphe: 4.8 ± 3.5, lateral hindbrain cluster: 7.6 ± 4.0, mean ± s.d.) (Fig. 5h, Methods: Anatomical enrichment). Additionally, we quantified the diversity of QEM-1 and wake encoding neurons by determining optimal cluster numbers (Supplementary Fig. 13).

Strikingly, the activity of many QEM-1 state-encoding neurons was not a simple binary indicator function of behavioral state. Instead, they exhibited both positive and negative ramping dynamics that spanned the duration of each QEM-1 period (Fig. 5i–k). We fit exponential curves to each neuron’s QEM-1 activity to quantify the dynamics of these ramps (Methods: Exponential Model Fits to QEM-1 Activity). Ramping shapes were stereotyped according to anatomical location (Fig. 5i–k). In both the inferior and dorsal raphe, most neurons (i.e., the “ramp down neurons”) were best fit by exponential decay and decay time constants were significantly larger in the dorsal raphe than in the inferior raphe (dorsal raphe: τ = 0.91 ± 0.03 arb. units, inferior raphe: τ = 0.67 ± 0.03 arb. units, mean ± s.e., *p* = 1.3 ×10^-6^, rank sum test; note: the duration of each QEM-1 period was rescaled to range from 0 to 1). Conversely, lateral hindbrain cluster neurons were best fit by “1 minus exponential” dynamics (ramp up neurons) with relatively short time constants (τ = 0.21 ± 0.01 arb. units, mean ± s.e.). Notably, the ramp up neurons were not as well fit by the exponential models in general (R^2^ for hindbrain ramp up neurons: 0.05 ± 0.06 vs. dorsal raphe neurons: 0.17 ± 0.16 and inferior raphe neurons: 0.23 ± 0.17, mean ± s.d.). Instead, many neurons showed stochastic activation with increasing probability towards the end of QEM-1 (Fig. 5j).

We note that in addition to saccade-encoding and QEM-1 state-encoding neurons, there are also neurons with spontaneous activity uncoupled to either behavioral or internal state variables. Across the brain, we extracted the rate of transient neural activity events for each neuron during each state (Methods: Spontaneous neural activity events, Fig. 5o) and identified neurons exhibiting a high frequency of phasic firing events ( > 0.014 events/s) during both wake and QEM-1 (Fig. 5p). These spontaneously active neurons are anatomically enriched in the zebrafish telencephalon (Fig. 5q, Supplementary Fig. 15a–d), which includes neural populations potentially analogous to the mammalian hippocampus and cortex.

### QEM-1 evolves in time according to stereotyped neural dynamics

Given the complex encoding of QEM-1 state by individual neurons, we performed whole-brain PCA to gain a more comprehensive view of global dynamics during QEM-1 (Methods: PCA of whole brain activity). Contrary to the fixed-point attractor model of sleep reported in *C. elegans*^57^, we observed substantial neural dynamics within QEM-1 itself. To investigate this, we restricted our whole-brain PCA analysis to QEM-1 timepoints only (Fig. 5l, Methods: PCA of whole brain activity). In the resulting QEM-1 PC space, activity evolved in an organized fashion throughout time during QEM-1 (Fig. 5l). This was consistent across fish (Supplementary Fig. 14a). Performing the same analysis on wake periods, we found qualitatively similar results (Fig. 5m, Supplementary Fig. 14b). However, wake trajectories appeared to be less organized, perhaps reflecting the diversity of the waking brain state. Rather than exhibiting a distinctive geometry, wake state-space trajectories were often overlapping (i.e., “tangled”)^58^. Indeed, temporal entanglement was significantly lower during QEM-1 than during wake (Fig. 5n, *p* = 1 ×10^-3^, *n* = 11 fish, Wilcoxon signed rank test, Methods: entanglement of state space trajectories). This suggests that the highest variance dimensions of QEM-1 activity can be reliably used to decode time progression, while the highest variance dimensions of wake activity also reflect other variables in addition to internal state, such as motor activity. Thus, we conclude that neural activity during the QEM-1 state is primarily dominated by neuronal populations with slow ramping dynamics. Collectively, these populations produce disentangled representations of time in the population activity state space.

**Fig. 6:**
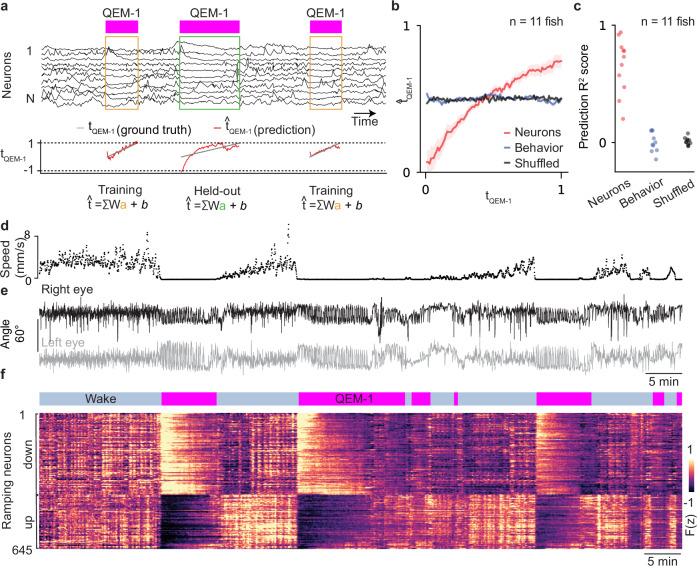
Decoding relative time progression in QEM-1 from brain-wide neural dynamics. **a** Relative time decoding procedure. Top, QEM-1 state labels and activity of 11 example neurons smoothed with a Gaussian kernel (σ = 25 s). The linear decoder was trained on all QEM-1 periods except for one held-out period. Trained decoder weights were then applied to the held-out QEM-1 period to predict relative time. This procedure was repeated for each QEM-1 period in order to generate predictions for the entire dataset. Bottom, relative time prediction (red) in each QEM-1 period compared with true relative time (gray). **b** Relative time prediction (mean ± s.e.) using only neural activity (red), behavioral variables (blue), or shuffled neural activity (black) (*n* = 11 fish). **c** Decoder performance was quantified by measuring R^2^ between true relative time and predicted relative time (neural R^2^ = 0.66 ± 0.07, shuffled R^2^ = 0.01 ± 0.01, behavior R^2^ = -0.03 ± 0.04, mean ± s.e., *n* = 11 fish). **d**,**e** Speed (mm/s) (d), left eye (**e**, gray), and right eye (**e**, black) angle of an example freely swimming larva. **f** Top, behavioral state labels (blue: wake, magenta: QEM-1) throughout the experiment. Bottom, raster map of z-scored neural activity for neurons in the example animal that significantly contributed to relative time decoding during QEM-1 state: ramp-down neurons (388 neurons, upper rows), which had consistent negative decoding weights across QEM-1 periods, and ramp-up neurons (257 neurons, bottom rows), which had consistent positive decoding weights.

Whole-brain PCA results (Fig. 5l–n) demonstrated that neural activity evolves stereotypically during QEM-1. This insight motivated us to try decoding time progression in QEM-1 from neural activity. To this end, we trained a regularized linear decoder (Supplementary Fig. 16a, b, Methods: Relative time decoding from whole-brain activity) to predict relative time within the QEM-1 state using whole-brain neural activity (Fig. 6a). To avoid overfitting, we evaluated decoder predictions on held-out QEM-1 states. Therefore, we restricted our analysis to datasets with at least two QEM-1 periods. Neural activity predicted relative time progression through the QEM-1 state accurately (Fig. 6b, c; *R*^*2*^ score: 0.66 ± 0.07, shuffled control *R*^*2*^ score: 0.01 ± 0.01, mean ± s.e., *p*-value = 9.7 ×10^-4^, Wilcoxon signed rank test, *n* = 11 fish). The decoder utilized both ramp up and ramp down neural populations, which were enriched among QEM-1-encoding neurons (Fig. 6d–f, Supplementary Fig. 16c, d, Methods).

It is possible that the neural activity that was predictive might actually reflect subtle, state-dependent dynamics in behavior across time (e.g., saccade rate, roll, pitch, speed, eye angles), instead of internal state. To test this possibility, we trained an additional decoder to predict relative time using behavioral metrics alone (Methods: Relative time decoding from whole-brain activity). Unlike neural activity, behavior alone was not able to predict relative time in QEM-1 (Fig. 6b, c, behavior control *R*^*2*^ score: -0.03 ± 0.04, mean ± s.e., *n* = 11 fish). This confirms that neural dynamics evolve in time according to the progression of internal state variables and are not simply a reflection of observable motor patterns.

Relative time decoding during QEM-1 motivated us to investigate whether there are also latent dimensions of awake neural activity that encode time progression during wake state. To test this, we employed the same linear decoding strategy to decode relative time progression in the wake state (Supplementary Fig. 17a). Although decoding performance was slightly lower on average than for QEM-1, time progression during wake could also be predicted from neural activity alone (Supplementary Fig. 17b, c, R2 score: 0.60 ± 0.05, shuffled control *R*^*2*^ score: -0.01 ± 0.01, mean ± s.e., *p*-value = 9.7 ×10^-4^, Wilcoxon signed rank test, *n* = 11 fish). Across fish, wake ramp-up and ramp-down neural activity contributed to time decoding (Supplementary Fig. 17d–f). Unlike QEM-1, time progression in wake, for some fish, could be decoded above chance levels using behavior alone (Supplementary Fig. 17b–e, behavior control *R*^*2*^ score: 0.12 ± 0.06, mean ± s.e.).

Collectively, these results suggest that QEM-1 and wake are not fixed points in state-space that transition stochastically back and forth but, instead, follow stereotyped dynamical trajectories across time within each state.

## Discussion

Previous studies in zebrafish have reliably identified sleep periods during both day and night by locomotor quiescence (lasting ≥1 min) which are associated with elevated arousal thresholds^3,23,25,26,29,30^. Here, we extend this operational definition by performing the first systematic investigation of eye movement kinematics during naturally occurring sleep states across the circadian cycle in freely moving larval zebrafish. The kinematics of eye movements provide a robust behavioral metric that further partitions zebrafish sleep into distinct substates – QEM-1, QEM-2, QEM-3, and QNEM. Thus, contrary to earlier reports in immobilized preparations^13^, where experimental constraints may have obscured core features of vertebrate sleep behavior, eye saccades are a prominent feature of spontaneous sleep in freely behaving fish.

A central implication of these findings is that sleep with eye movements is not a unitary state. Whereas mammalian sleep groups eye-movement–associated sleep into a single REM state, larval fish express multiple sleep substates that differ in eye-movement kinematics and show distinct circadian patterns of substate occupancy. Daytime sleep is dominated by QEM-1, whereas nighttime sleep increasingly engages QEM-2 toward morning, revealing a sleep architecture that extends beyond a simple REM/NREM dichotomy.

Brain-wide imaging during QEM-1 provides a mechanistic foothold into the neural dynamics that underlie a novel sleep substate. QEM-1 is accompanied by broad suppression of brain activity, including reduced activity in the noradrenergic locus coeruleus. However, a small population of neurons remain active and encode elapsed relative time within QEM-1 through stereotyped trajectories in low dimensional state-space. Thus, rather than collapsing to a fixed point in population activity^57^, QEM-1 state-encoding neurons maintain an internal temporal structure within each QEM-1 period. Extending this approach to the remaining substates will require more light-efficient tracking microscopes that can sustain long recordings without perturbing sleep substate expression, particularly during the night.

Our results establish eye-movement kinematics, integrated with locomotion, as a simple, scalable behavioral readout that reveals a conserved, multi-state sleep architecture in closely related *Danio* species. Four discrete substates emerge, distinguished not only by the presence or absence of eye-movements, but also by the kinematic structure of conjugate saccades. Circadian phase and ambient light jointly gate substate expression, defining a compact control logic for sleep-state selection. This provides both an advanced framework for how vertebrate sleep can be organized outside mammals and a foundation for dissecting the neural dynamics and functions of individual substates.

## Methods

### Behavioral imaging at high spatiotemporal resolution across many animals

We developed a new behavioral acquisition system called DASHER (Dynamic Addressable System for High-speed and Enhanced Resolution), which combines real-time animal tracking with deep image sensor integration to achieve enhanced spatiotemporal resolution with a variety of commercial image sensors to significantly boost spatial and temporal resolution around the animal of interest. Briefly, two versions were used for behavioral experiments, a version optimized for detailed behavioral imaging of a single animal with a commodity USB3 camera, and a version optimized for detailed behavioral imaging of up to 20 animals with a higher bandwidth 25GigE camera.

In the single fish version of DASHER, we use a USB3 Blackfly S BFS-U3-51S5M-C (FLIR) with a Sony IMX250 sensor. We configure the image sensor to read out only a small region of interest (ROI) encompassing the animal and its immediate surroundings (e.g., static environment such as walls or dynamic elements such as live prey), thus significantly increasing the imaging framerate without sacrificing the overall field of view (FOV) or the spatial resolution of the ROI around the animal. As the animal moves, DASHER performs a live update of the left and top readout offsets of the image sensor in the camera, such that the ROI is dynamically updated to remain centered on the animal. Animal tracking is adapted from our previous publication^52^, using CUDA to accelerate a brute-force search over all positions (x, y) and heading angles (θ) for the best match (Pearson correlation) to a masked template image derived from the same animal, with the tail removed. By using DASHER to address only a small portion of the sensor, we increase the framerate of the Blackfly S camera roughly 4-fold, allowing us to achieve an imaging rate of 250 Hz with a spatial resolution of 20 μm and a FOV of 10.24 × 10.24 mm (512 × 512 pixels). We use this version for behavioral recording in arousal and neural datasets. Strobed near-infrared (NIR) and white light illumination was previously described^31^.

In the multi-fish version of DASHER, we used a large sensor camera with a high bandwidth 25 gigabit per second (Gbps) interface (Emergent Vision Technology HB-25000-SB, 5328 ×4608 at 50 frames per second) to simultaneously image up to 20 animals per experiment across 24 h. Each animal is placed into a separate circular chamber (28 × 28 × 1 mm). An ROI (256 × 256 pixels, corresponding to 12.8 × 12.8 mm at 50 μm/pixel) was maintained around each animal by running 20 instances of the same GPU-based fish tracker as above, and then these ROIs were streamed to disk. A large rectangular PCB (280 × 240 mm) with interleaved NIR (SFH4650-Z, OSRAM) and white (GW PSLM31.FM OSRAM) LEDs was placed below the behavioral chamber. We use this version for behavioral recording at circadian timescales.

### Eye-tracking

Each NIR zebrafish image was transformed into an egocentric view using the tracked fish position and heading. In the egocentric view of the fish, only a small region of interest ( ~ 0.9 × 0.9 mm) around the head was used for further processing. Next, we applied a difference-of-Gaussian (DoG) filter to the region of interest to isolate the eyes and suppress background noise (single fish DASHER: σ₁ = 0.10 mm, σ₂ = 0.06 mm; multi-fish DASHER: σ₁ = 0.15 mm, σ₂ = 0.07 mm). We defined the *y* position of the eyes as the peak of the maximum projection of pixel intensities onto the body axis. The *x* position of each eye was computed by projecting pixel intensities onto the axis orthogonal to the body axis and fitting a bimodal Gaussian mixture model. After getting the center coordinates for the left and right eyes, we defined a rectangular mask around each eye and computed the central moments of each. From the central moments, we measured each eye angle.

### Saccade kinematics

Left and right eye angle traces were preprocessed by sequentially applying mean (200 s running window) and median filters (1.5 s running window) for noise reduction and then again filtered using a fused lasso filter (λ = 0.25). Saccades were defined as concurrent deflections of both eyes in the same direction exceeding a defined threshold. To this end, saccade candidates were first identified by detecting prominent peaks in the product of left and right eye velocities (single-fish DASHER threshold: 0.32 radians^2^/s^2^, multi-fish DASHER threshold: 0.60 radians^2^/s^2^, minimum inter-saccade interval: 1.5 s), computed from the filtered eye angles. Final saccade selection incorporated two criteria: interocular correlation >0.5 (5 s running window) and fused lasso residuals <0.45 radians (15 s running window) to exclude noisy saccade events.

For each identified conjugate saccade, we quantified two kinematic parameters: the decay slope, determined by applying linear regression to the raw eye angle from saccade offset until the amplitude decayed below 33% of the initial post-saccadic amplitude, and the fixation duration, defined as the interval between saccade offset and when the eye angle crossed the 33% amplitude threshold. Both parameters were calculated independently for each eye before being averaged to obtain binocular estimates.

### Sleep and QEM state classification

Locomotor speed was computed using a 5 s rolling-window average. Sleep periods were identified by applying a speed threshold ( < 0.5 mm/s), and only those lasting ≥1 min, consistent with established zebrafish sleep criteria^3,23,25,27^, were retained for analysis.

Sleep periods lasting ≥1 minute were divided into consecutive 1-minute bins. Any terminal segment ≤30 s was merged with the preceding bin, while remaining terminal segments between 30 and 60 s were treated as separate bins. For each bin, we quantified three parameters: (1) saccade count, (2) mean saccade decay slope, and (3) average fixation duration. Bins without saccades were classified as Quiescent with No Eye Movement (QNEM).

To categorize bins containing saccades, we fit a Gaussian Mixture Model (GMM; implemented via scikit-learn’s GaussianMixture function^59^, with the following parameters: n_components=3, covariance_type = “diag”, initial means set to a diagonally dominant matrix, and reg_covar=2×10^-5^) to the three parameters (saccade count, decay slope, and fixation duration) using the behavior data from the light/dark cycled condition. For model selection, we estimated the optimal number of components (3 components) using sample-size adjusted Bayesian information criterion (SABIC) which provides the optimal balance between model fit and complexity. The three components are distinguishable by their dominant features: QEM-1 showed the highest saccade frequency, QEM-2 exhibited the steepest saccade decay slopes, and QEM-3 was characterized by the longest fixation durations. After fitting, the resulting GMM was then applied to classify sleep substates across all experimental conditions.

### Artificial neural network (ANN) logistic regression model

The probability of each sleep substate (QEM-1, QEM-2, QEM-3, QNEM, and Wake) was modeled as a function of three input variables, each of which we qualitatively determined to have a large impact on state emission probabilities. The input variables were 1) luminosity, 2) time of introduction to chamber, and 3) circadian timing. Luminosity was an indicator variable (0 when the lights were off, 1 when the lights were on). Time of introduction was defined as 1 on the day that animals were loaded into the experimental chamber, and 0 for the remainder of the experiment. Circadian time was defined as a cosine wave and a sine wave, each with a period of 24 hours and an offset based on the time of day that the experiment started. Thus, the combination of the sine wave and cosine wave together provided a circular representation of time within the circadian cycle.

In order to model the potentially nonlinear relationship between the input variables and the output, we used an artificial neural network in PyTorch to implement a logistic regression model that transforms these three inputs into state probabilities^60^. The model consisted of three layers: An “encoder” layer with 24 hidden units, a 1-dimensional latent layer, and a “decoder” layer with 24 units. Each layer utilized sigmoid activation functions. The output of the decoder layer was fed into a logistic regression output layer with five units, each representing the probability of one of the five behavioral states. The output of these five units was constrained to sum to one.

The model was trained using fish behavior data from all 24-hour experiments (including light cycling, constant light, and constant dark). To estimate confidence intervals for the state probabilities output by the model, we performed 5-fold cross-validation with 20 repetitions. Thus, the model fitting procedure was repeated 100 times, each time using a unique bootstrapped sample of the full dataset, yielding an ensemble of 100 individual models. During each iteration, the model was trained on data from 80% of the fish. Predicted state probabilities were generated for each experimental condition: light cycle, constant light, and constant dark. For each model in the ensemble, performance was evaluated by measuring the correlation coefficient between the model-generated and experimentally observed state probabilities in the 20% of held out fish. We used n-fold cross validation to ensure that the model was evaluated on all data. This was repeated 10 times in order to reliably estimate model confidence intervals.

Finally, to quantify the impact of each input variable on predicting state probabilities, we performed ablation studies in which one of the three input variables was time shuffled prior to model fitting. The reduction in model performance associated with the shuffling of a variable indicates that variable’s contribution to predicting behavioral state.

### Optomotor response to moving gratings

To verify that *lakritz -/-* mutants were indeed blind, we tested their optomotor response to moving grating stimuli using multi-fish DASHER. Fish were allowed to swim freely in 40 × 20 mm behavioral chambers. A projector (Optoma HD29H) was used to display a moving, full contrast (black/white) grating (spatial period 3–10 mm) for 15 s every 45 s over the course of a 1-hour experiment. Behavioral responses were quantified by applying a Gaussian smoothing kernel (σ = 1 s) to the animal’s velocity, where positive velocity is defined to be in the direction of the moving grating during the 15 s stimulus presentation. In one hour, 100% of control *lakritz +/+; mitfa-/-* fish (*n* = 5 fish) and 0% of *lakrtiz -/-* mutants (*n* = 11 fish) responded to the gratings.

### Dark flash arousal assay

We delivered full-field dark flash stimuli that are known to elicit high amplitude escape turns^40^. Each experiment lasted two hours. Full-field dark flash stimuli lasting 1 s each were presented by turning off all visible white light in the arena once every 60 s for a total of 120 stimuli per experiment. Tail tracking was performed (see below) and the angle of the tip of the tail, relative to animal heading, in the 1 s response window (time during lights off) was analyzed. Within the 1 s response window, the first time point with a relative tail angle amplitude greater than 180 degrees was counted as a dark flash response. Response probability was computed separately for each sleep substate.

For the randomized dark flashes, we delivered full-field dark flashes with inter-stimulus intervals (ISI) drawn from a truncated normal distribution with a mean of 120 seconds and standard deviation of 90 seconds (lower bound: 75 seconds, upper bound: 165 seconds). The average duration of randomized trial experiments was 2 hours.

### Tail tracking

Tail tracking was achieved using an optimization-based algorithm described previously^31^. Briefly, the algorithm created a high-resolution spline to trace out the curvature of the tail for each NIR image of the animal. The spline consisted of a series of 100-μm long straight segments (5 pixels) with a parameter for local angle change at each segment boundary. A nested grid search was used to fit the optimized tail spline onto the fish tail. The algorithm was designed to achieve robust tracking even in the presence of bright artifacts near the tail as previously described. The number of tail segments was overprovisioned during fitting and then trimmed post-hoc to the first 28 tail segments, in order to define a robustly tracked point near the tip of the tail at the given camera scale and animal age.

### Mechano-acoustic tap arousal assay

A solenoid was mounted to the plate holding the fish chamber. Every 2 min, the solenoid tapped the plate, providing mechano-acoustic stimulation to the fish through the coupling of the glass chamber to the metal plate. Experiments lasted between 2 and 5 h. Startle responses to the acoustic tap were defined as moments where the instantaneous speed of the fish in the 1 s window following the tap stimulus exceeded 150 mm/s. Response probability was computed separately for each sleep substate.

For the randomized tap stimuli, we delivered mechano-acoustic taps with inter-stimulus intervals (ISI) drawn from a truncated normal distribution with a mean of 120 s and standard deviation of 90 s (lower bound: 75 s, upper bound: 165 s). The average duration of randomized trial experiments was 2 h.

### Daytime sleep deprivation

In order to deprive fish of daytime sleep, we designed a 6-hour visual stimulus protocol using full contrast (black/white) drifting gratings which are known to elicit robust directional swims in zebrafish larvae. Using an Optoma HD29H projector, we projected full contrast, whole field, drifting gratings onto the floor of our glass behavioral chambers. In order for an image to be formed on the floor of our glass chambers, we sandblasted the bottom of the glass to create an opaque surface on which the black/white bars of the grating could clearly be seen.

The stimulus protocol consisted of 15 s of moving gratings, followed by 15 s of neutral gray screen. The direction of motion of the gratings was changed (left / right / up / down) each time the stimulus was presented to prevent fish from adapting to the stimulus. This protocol was repeated for 6 hours. During each experiment, we divided the batch of larvae into treatment and sibling control fish. Treatment fish experienced the described moving grating stimulus, while control fish experienced a gray screen with the same average luminosity as the moving gratings.

### Water flow experiments

To test whether water flow periods can induce QEM-1, we recorded behavior using single-fish DASHER while animals experienced water flow in a perfusion chamber. We used an elliptical PDMS chamber (30 × 20 mm). An initial 6 min behavioral baseline was recorded without water flow. Subsequently, water flow ( ~ 2.5 ml/min) was introduced for 3 min. Following the water flow period, behavior was recorded for an additional 6 min. Control experiments of equivalent duration were conducted without water flow. The QEM-1 state probability during pre- and post-flow periods was compared to assess the effects of water flow on QEM-1 induction.

Flow experiments were also performed on the tracking microscope in order to simultaneously record behavior and neural activity. In these experiments, an initial 6 min of baseline activity was recorded without water flow, followed by 1 min of water flow and then an additional 10 min without flow. For the analysis shown in Supplementary Fig. 11, neurons that were effectively sampled at <1 Hz or that were inactive (fluorescence distribution with mean <median) during a 3 min window around flow (1 min of flow ± 1 min) were excluded.

For longitudinal experiments spanning 48 h with 5–8 dpf larval zebrafish (multi-fish DASHER), we designed a custom array of three chambers, arranged vertically (28 × 28 × 1 mm per chamber) and connected via narrow slits (height <1 mm) in the PDMS that permitted water flow but prevented larval passage. These slits were reversibly sealed with movable magnets of smaller height ( < 1 mm) than the slits to maintain chamber isolation while allowing continuous water exchange. The chamber array had an inlet at the top connected to a peristaltic pump delivering E3 medium at ~0.5 ml/min, and an outlet at the bottom, maintaining gentle flow conditions that did not perturb natural fish behavior.

### Simultaneous behavioral and neural imaging of larval zebrafish

Fish were allowed to swim freely in a circular behavior arena (28 mm in diameter and ~1 mm in height) as described in previous work^31^. The chamber was illuminated with white light delivered either to all sides of the behavioral arena or from above. For behavioral tracking, the arena was illuminated by four custom NIR light strips with narrow angle, 850 nm LEDs (SFH4655-Z, Osram) that directed infrared light into the chamber.

To image neural activity, we conducted tracking DIFF microscopy experiments as previously described^52^ but with an updated motion cancellation system that utilizes a custom 3 axis motorized stage. We imaged brain-wide calcium activity within a volume of 1014 × 764 x 150 μm at cellular resolution in freely swimming fish expressing pan-neuronal H2B-GCaMP6s or H2B-GCaMP8s at 2 volumes/s. The details of this system have been described previously^52^. A 2-stage hierarchical HMM classified zebrafish behavior into waking, sleep substates using speed and saccade rate, respectively. To reduce overfitting artifacts, posterior probabilities were Gaussian-smoothed before Viterbi decoding.

### High-resolution offline registration of fluorescent brain volumes

We matched each fluorescence image from the tracking microscope for each animal to a high-resolution reference brain volume collected from the same animal. The registration pipeline, which includes a 3D rigid transformation and a non-rigid transformation, was described previously^52^. Briefly, each individual DIFF-sectioned brain slice (the moving image) is aligned to a plane within the reference brain volume with a 3D rigid transformation. Registration is then refined by locally adjusting a subdivided deformable surface within the reference volume using a regularized piecewise affine transform.

Parameters extracted from the affine registration step were used to compute fish pitch and roll during each time point (Supplementary Fig. 9).

### Non-negative matrix factorization and post processing

After image registration, we extracted raw fluorescence *F*(*t*) across time (*t*) for each neuron in the brain using non-negative matrix factorization^61^ (NMF, *n* = 11 fish, 114,435 ± 21,267 neuronal centroids, mean ± s.d., per fish). NMF decomposes the recorded spatiotemporal image data into two factors – one containing the spatial footprint of each neuron and another containing the time-varying raw fluorescence of each neuron. We applied spatially constrained NMF to our whole-brain datasets. NMF was performed independently for each axial section of a given brain volume. To avoid redundant neuronal centroids, we merged ROIs which were likely to represent repeated sampling of the same neuron across multiple axial planes. We did this by identifying and excluding ROIs which were 4 µm or less apart from each other in all the three dimensions and were also highly correlated ( > 0.8 correlation coefficient).

Due to free swimming fish behavior, each neuron could remain unobserved in any given brain sweep (e.g., because it fell outside the axial scan range of a given sweep). Therefore, we only included neurons for further analysis whose raw fluorescence traces had at least 50% available data, indicating that the neuron was tracked well over the course of the experiment (i.e., an overall sampling rate of at least once per second). After this step of preprocessing, we were left with 74,215 ± 23,457 (mean ± s.d.) neurons per fish. Missing values in the raw fluorescence traces were linearly interpolated for all analyses that relied on continuously available data traces (whole brain PCA, time decoding from neural activity).

Finally, to account for changes in fluorescence baseline due to photobleaching, we fit an exponential decay to the fluorescence timeseries of each neuron to estimate slow timescale bleaching. To correct for this, we subtracted the estimated bleaching from the neuron’s raw fluorescence.

### Registration to a common reference brain across animals

We registered our live imaging data (moving image) to a reference atlas zebrafish brain (mapZebrain atlas^62^) using CMTK^63^. Registration was performed in three steps. First, an initial affine transformation was defined to match the center of mass of our live data with the atlas brain. Next, we fit a full affine transformation (translation, rotation, scale, and shear). Finally, a non-rigid warping was applied to the affine transformed brain which maximized normalized mutual information between our brain and the atlas brain. We optimized the parameters of this fit to preserve cell morphology while achieving the best possible global alignment between our brain and the atlas brain. After testing many different parameter combinations, we arrived at the following procedure for registration:

Step 1: cmtk make_initial_affine --centers-of-mass ATLAS.nii FLOATING.nii initial.xform

Step 2: cmtk registration --initial initial.xform --dofs 6,12 --ncc --exploration 50 --accuracy 3 -o affine.xform ATLAS.nii FLOATING.nii

Step 3: cmtk warp -o nonrigid.xform --nmi --jacobian-weight 0 --accurate -e 18 --grid-spacing 256 --energy-weight 1e-1 --refine 3 --coarsest 4 --ic-weight 0 --output-intermediate --accuracy 3 --initial affine.xform ATLAS.nii FLOATING.nii

The resulting nonrigid.xform transformation was used to transform our NMF coordinates into the reference brain atlas space, allowing us to assign brain region labels to our data and pool results across all fish.

### Multivariate linear regression analysis

We used multivariate linear regression to regress the activity of each neuron against 9 timeseries variables (six behavioral variables and three QEM-1 state-related variables). Behavioral variables included speed, roll, pitch, left eye angle, right eye angle and turn bias (defined below). QEM-1 state variables included a binary indicator function for QEM-1 state, its integral, and its positive derivative (convolved with an exponential kernel, time constant = 1/15 * the duration of the QEM-1 period). Collectively, these vectors comprise a 9-by-time matrix, *X*, such that we can define our model for a given neuron, *y*_*i*_ as:

1 $${y}_{i}={X}^{T}{\beta }_{i}+{\beta }_{0}$$

where *β*_*i*_ is the 9-element column vector of regression coefficients and *β*_*0*_ is a baseline vector for this neuron. We fit each cell using 10-fold nested cross validation. Briefly, data was divided into 10 non-overlapping time windows. Fits were performed on 90% percent of the data and evaluated on the held-out 10% percent. This was repeated 10 times to generate a prediction of the entire time series. Model performance was quantified using *R*^*2*^ between the predicted and actual activity in the held-out validation data. To test the significance of each specific regression variable, we fit additional models, using the same procedure, but in which regressor(s) of interest were temporally shuffled prior to model fitting. This procedure effectively broke the temporal relationship between the regressor and the neural activity, while preserving the number of degrees of freedom in the model. Thus, we could compare *R*^*2*^ of the shuffled model fit directly to the full model. A regressor was deemed to contribute significantly to the variance of a neuron if, after shuffling, the reduction in mean *R*^*2*^ across validation sets was larger than 2 times the standard error of *R*^*2*^ across validation sets. We ran three such partial models: 1) left / right eye angles shuffled, 2) turn bias shuffled, 3) QEM-1 variables shuffled. We used these models to identify the neurons encoding eye position, turn bias, and QEM-1, respectively.

### Definition of turn bias

We defined a behavioral regression variable based on animal heading in order to capture oscillatory turning dynamics. Turn bias was defined by convolving fish heading angle change with a Gaussian kernel (σ = 8 minutes). This resulted in a smoothed version of Δheading that remained stably positive or negative over the course of multiple minutes, reflecting sustained turn direction bias (Supplementary Fig. 12).

### In situ labeling of dbh positive cells

We performed imaging in freely moving fish as described above. We used chambers with confined areas (e.g., corners) as these were found to induce QEM-1 more reliably. We fixed and labeled fish with in situ hybridization^25,54^. Briefly, we first fixed the fish in 4% PFA in PBST at 4 °C overnight, then incubated for 2 to 10 min in methanol at −20 °C, followed by rehydration with 50% and 25% methanol for 5 min each. We then incubated the fish overnight at 37 °C with DNA probes designed to tile the dbh mRNA (Molecular Instruments, Inc). We then washed these probes and incubated overnight at room temperature with fluorescently labeled snap-cooled hairpins with an excitation wavelength of 514 nm (B2 amplifier, Molecular Instruments, Inc.). Finally, we removed the hairpins with 4 washes (2 × 5 min, 2 × 30 min and 1 × 5 min) in 5X SSCT. We mounted the fish in 2% agarose and imaged the fluorescent labeling as a brain volume with a LSM 780, AxioObserver confocal microscope (Zeiss) with a LD LCI Plan-Apochromat 25x/0.8 Imm Korr DIC M27 objective at 1 × 1 × 2 µm resolution.

We used CMTK to align the confocal GCaMP stack with the averaged functional stack: Step 1: cmtk make_initial_affine --centers-of-mass Functional.nii Confocal.nii initial.xform Step 2: cmtk registration --initial initial.xform --dofs 6,12 –ncc or nmi --exploration 75 --accuracy 4 -o affine.xform Functional.nii Confocal.nii Step 3: cmtk warp -o nonrigid.xform --nmi, smi or ncc --jacobian-weight 0 --accurate-e 18 --grid-spacing 80 --energy-weight 1e-1 --refine 6 --coarsest 6 --ic-weight 0 --output-intermediate --accuracy 0.8 --initial affine.xform Functional.nii Confocal.nii

In cases for which the initial step didn’t give satisfying results, we preceded CMTK alignment with landmark registration using the “Name Landmarks and Register” plugin in FIJI. We applied the same transformation to the in situ staining data, and thresholded the resulting map to remove background and generate masks.

To extract single neuron traces from our functional data, we used a modified version of the NMF procedure described above. 2D NMF performed at 2 µm axial spacing leads to repeated sampling of cells that span more than one axial plane. Thus, multiple axial ROIs can correspond to a single cell in the in-situ patterns. To resolve this, we instead separated the volume in sub-volumes of 31×56×56 pixels overlapping by 6 pixels in *x*, *y* and *z*. We first processed these volumes using PCA/ICA as described^64^ which led to maps with well separated cells in 3D. We used these maps to obtain cell centroids (local maxima), and NMF to unmix the volume in the vicinity (6 µm) of these centroids as described above.

To compare the activity between wake and QEM-1, we averaged single neuron z-scored traces for each behavioral state, and then averaged each value per fish.

### Anatomical enrichment

To determine which brain regions were enriched for QEM-1 encoding neurons, we computed the fold-enrichment of QEM-1 neurons in each brain region relative to a random sample of neurons in the brain. That is, for a given brain region, *X*, we first determined the number of QEM-1 positive neurons, *n*_*pos, region*_, and the number of total neurons imaged in the region, *n*_*total, region*_. These were used to define the proportion of QEM-1 neurons in this brain region, *P(X)*.

2 $$P\left(X\right)=\frac{{n}_{{pos},{region}}}{{n}_{{tot},{region}}}$$

Next, we sampled *n*_*pos, region*_ neurons from the pool of all recorded neurons in the experiment, across the entire imaging volume, and determined how many of these neurons were QEM-1 positive, *n*_*pos, rand*_. We generated a null distribution, *P*_*0*_*(X)* by repeating this procedure 100 times.

3 $${P}_{0}\left(X\right)=\frac{{n}_{{pos},{rand}}}{{n}_{{tot},{region}}}$$

Finally, fold enrichment, *E(X)*, was defined as the ratio of actual probability that a neuron in region *X* is QEM-1 positive to the null probability of finding a QEM-1 positive neuron in the brain.

4 $$E\left(X\right)=\frac{P\left(X\right)}{{P}_{0}\left(X\right)}$$

For a given fish, we defined regions as significant if *P(X)* > *P*_*0*_*(X)* in at least 95% of random samples (p < 0.05). Across fish, a region was declared significant if at least 8/11 fish met this criterion. We ran this analysis for all brain regions available in the MapZebrain atlas as well as a custom defined region in the hindbrain based on the results in Fig. 5e.

### Exponential model fits to QEM-1 activity

We used exponential model fits to quantify the dynamics of positively modulated QEM-1 neurons within each QEM-1 period. QEM-1 states with durations <3 min were excluded from this analysis. First, the activity of each identified QEM-1 neuron was linearly interpolated to remove missing values. Next, a QEM-1 “trial”-by-time matrix was built by extracting the neuron’s activity during each QEM-1 period and resampling along the time axis to 1000 data points, thus representing activity across “relative” time within each QEM-1 state. Each QEM-1 state was then treated as a trial, and the mean across QEM-1 “trials” was computed and normalized, and the resulting 1000 timepoint vector was fit with exponential models. We fit two exponential models to each neuron, including an exponential decay model,

5 $$y\left(t\right)=a{e}^{\left(\frac{-1}{{bt}}\right)}+c;\left[0 < a < 1,0 < b < 2000\right]$$

and a “1 minus exponential” model,

6 $$y\left(t\right)=a\left(1-{e}^{\left(\frac{-1}{{bt}}\right)}\right)+{c;}\left[0 < a < 1,0 < b < 2000\right].$$

Models were fit by minimizing the squared error between true activity and predicted activity and performance was measured by *R*^*2*^. Individual neurons were classified as ramp up or ramp down according to which model performed better. No cross-validation was performed as our goal here was to categorize cells as ramp up or ramp down and to quantify their temporal dynamics.

### PCA of whole-brain neural activity

Neural activity was linearly interpolated and *z*-scored prior to performing PCA. In order to focus specifically on variance associated with QEM-1 or wake, respectively, we performed two analyses. In the first analysis, the activity during each QEM-1 period was extracted and time was downsampled such that each QEM-1 period was described by a neuron x 10 timepoint matrix. We computed the mean of these matrices across QEM-1 periods for each fish, performed PCA on this mean activity matrix, and projected the data from the full experiment onto the top two PCs of this space. In the second analysis we performed exactly the same procedure, but restricted to wake periods instead of QEM-1.

### Entanglement of state space trajectories

In order to measure how organized neural activity trajectories were in the PC space described above, we computed a measure of entanglement for each state trajectory. We defined entanglement as the number of times a given trajectory crossed over itself between the beginning and end of a given QEM-1 state. Self crossings were defined by first smoothing each QEM-1 trajectory (Gaussian kernel, σ = 12.5 s) and then changing from a discrete (neural data sampled at 2 Hz) to continuous time representation using linear interpolation between sampled timepoints. That is, each smoothed neural trajectory was represented by N-1 linear segments where N is the number of time points in the trajectory. For each linear segment, we determined how many times it intersected another segment from the collection. The total number of intersections was divided by 2 to count only unique crossings, and was then normalized by the duration of the given state trajectory. Measurements of QEM-1 entanglement were performed in the QEM-1 PCA space, and wake entanglement was measured in the wake PCA space.

### Relative time decoding from whole-brain activity

Population activity was used to decode relative time within each behavioral state (QEM-1 or wake). First, the activity of each neuron was linearly interpolated to remove missing values. Subsequently, *z*-scores were computed for the neural activity within each state, namely wake or QEM-1. For decoding, we excluded short state periods which lasted less than 3 min. To decode the relative time within a specific state, we subdivided the *z*-scored neural activity and behavioral metrics for each state into 100 bins, to represent the progression across relative time within each state. To establish the ground truth for relative time within the state, we defined a response variable that linearly increased from 0 at the beginning of the state to 1 at the end of the state. To predict this response variable, we applied an elastic net linear model (sklearn package in Python)^59^ to the population neural activity. This model incorporates L1 and L2 penalties as regularizers. During the training phase of the decoder, data from all instances of a given behavioral state, except one held out instance, were utilized. Relative time decoding performance then was evaluated on the held-out state instance. This prediction procedure was applied in turn to each state instance, and then after predicting relative time for all the periods, we employed a second stage of gain correction of relative time predictions. We fitted a common gain factor using the predictions of all except one held-out fish. We then gain-corrected the prediction of the held-out fish using the fitted gain factor. We repeated this for the predictions of all the fish.

An exhaustive search per fish was conducted to identify the optimal hyperparameters for both QEM-1 and wake states, as illustrated in Supplementary Fig. 16a, b. To evaluate the decoding performance against chance level models, we temporally shuffled the neural activity within a given state while preserving local temporal correlation. To determine whether a neuron consistently contributed to the decoding of relative time, we selected neurons whose median decoding weights across model fits were greater than 0 or less than 0. Because of the L1 component of model regularization, many neurons received a weight of exactly 0 during model fitting. Subsequently, we defined ramp up neurons as those having consistent positive decoding weights (median coefficients > 0) and ramp down neurons as those having consistent negative decoding weights (median coefficients <0) across different training sets.

### Spontaneous neural activity events

To detect transient events in neural activity, we first estimated the baseline activity for each neuron by computing the mean activity below the 10th percentile threshold (window size = 45 s) and subtracted this baseline from the raw neural activity to obtain baseline-subtracted signals. We then identified significant events by calculating the standard deviation of negative values (noise SD) as a measure of neuronal noise and defining an event detection threshold as the mean activity plus twice the noise SD. Candidate events exceeding this threshold were further refined by applying a minimum inter-event interval (6 s) and a minimum event duration (2 s) to eliminate spurious detections.

Event rates were quantified for each neuron in every recorded animal by dividing the number of detected events by the duration of each contiguous interval of a given behavioral substate (wake, QEM-1) and then averaging across all instances of the same substate. To obtain neurons with significant activity in both wake and QEM-1, we applied an event rate threshold ( > 0.014 events/s) for both wake and QEM-1.

For neuronal density visualization, we binned the horizontal and sagittal space (horizontal space bin size = 2 × 2 μm, sagittal bin size = 4 × 2 μm) and computed the density in these bins across fish registered to a reference brain volume. These bins were subsequently smoothed using a Gaussian kernel (σ = 2 × 2). For quantifying enriched brain regions which were active during both wake and QEM-1 states, we employed the same anatomical enrichment method described above. Across fish, brain regions were designated as significant if they showed significant enrichment in at least 8 out of 10 fish.

### Statistical analysis

Sample sizes, statistics, and statistical tests are reported in the figure legends and main body of the results. For comparisons of behavior and neural activity across different experimental or behavioral state conditions, we used Wilcoxon rank sum and Wilcoxon signed rank tests. In all cases, two-sided tests were performed. In all cases where multiple observations (e.g., neurons) were collected from one animal, we first computed the mean statistic within each animal before performing the statistical test across conditions. While this approach slightly reduces statistical power, it leads to more conservative estimates of *p*-values. Sample size was not predetermined by a statistical method. The experiments were not randomized and were not performed with blinding.

### Animal care and transgenic lines

Experiments were performed in accordance with the Animal Welfare Office at University of Tübingen and the Regierungsprasidium. The majority of the imaging experiments were performed using Tg (elavl3:H2B-GCaMP6s + /+ or elavl3: GcaMP6s + /+) with *nacre* (*mitfa*-/-) at 5-8 dpf. In a subset of imaging experiments, we used Tg (elavl3:H2B-GcaMP8s + /+) with *nacre* (*mitfa* -/-) at 5-8 dpf. Behavioral experiments utilized Tg (elavl3:H2B-GcaMP6s + /+ or elavl3: GcaMP6s + /+), *nacre* (*mitfa* -/-), Tg (*ennife* -/-), *D. rerio* AB strain, *D. rerio* wild-type (+/+), *D. aesculapii* wild-type (+/+), and *D. nigrofasciatus* wild-type (+/+) at 4-8 dpf. All larval fish were reared on a 14/10 hr light/dark cycle at 28 °C. They were maintained in groups of 20 and fed dry food daily during the rearing period. During behavioral recording sessions, fish were not fed.

### Reporting summary

Further information on research design is available in the Nature Portfolio Reporting Summary linked to this article.

## Supplementary information

Supplementary Information

Description of Additional Supplementary Files

Supplementary Video 1

Supplementary Video 2

Supplementary Video 3

Supplementary Video 4

Supplementary Video 5

Reporting Summary

Transparent Peer Review file

## Source data

Source Data

## Data Availability

Source data for all graphs generated in this study are provided as a Source Data file with this paper. Raw imaging data are available from the corresponding authors upon request due to their large file size ( > 20TB). Source data are provided with this paper.

## Code availability

Code used to generate the findings and figures of this study is available at https://github.com/crheller/saccade-ms.git.
