# Supplementary material for: Eye movement kinematics reveal novel circadian organization of sleep substates
Source: Nat Commun. 2026 May 5;17:4068. doi: 10.1038/s41467-026-72222-0 (PMC13144723; doi:10.1038/s41467-026-72222-0)
Supplement: Supplementary file 1 — Supplementary Information [file 41467_2026_72222_MOESM1_ESM.pdf]

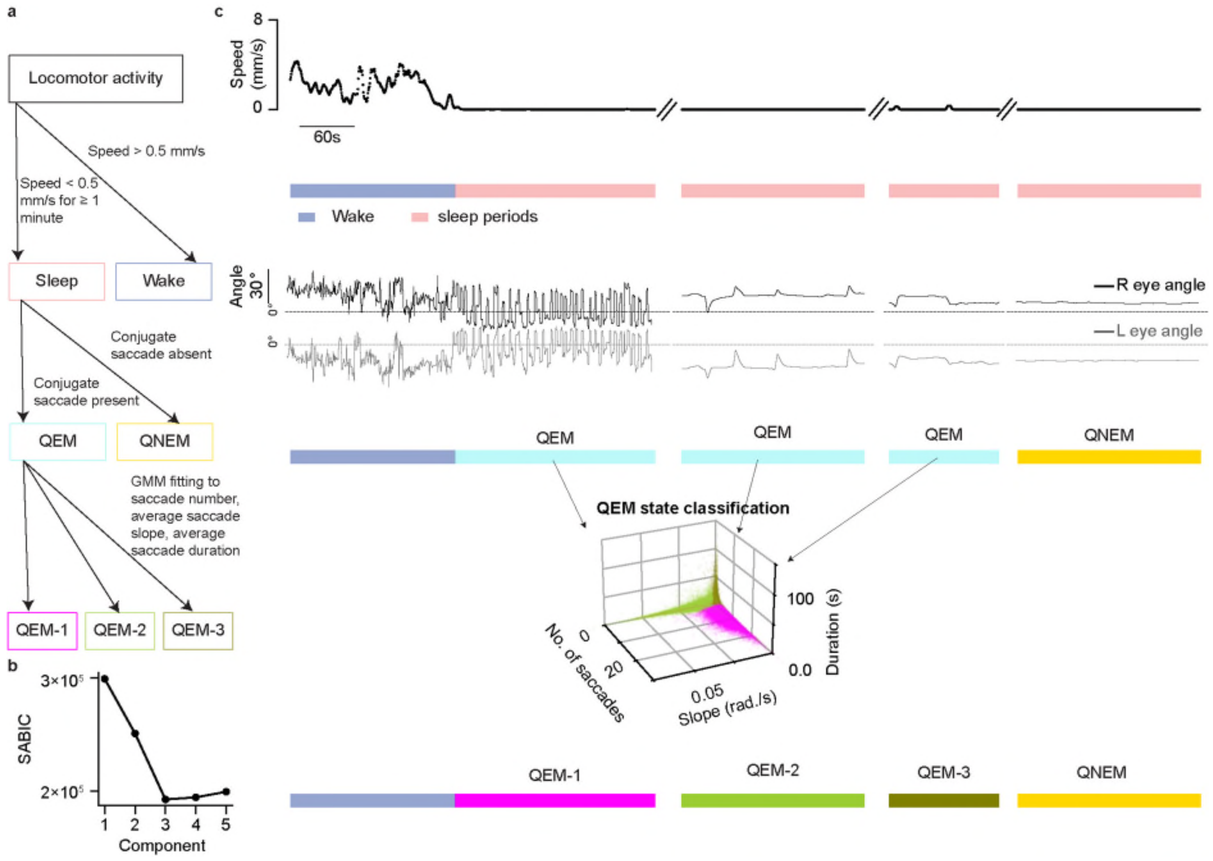

Supplementary Figure 1 | Sleep substate labelling workflow based on locomotor activity and eye movement Kinematics. a, A flowchart for identification of sleep substates. First, we classify sleep periods ≥ 1 min and wake periods (speed > 0.5 mm/s). Next, we examine 1-min bins during the sleep periods, identified previously, to determine whether there were conjugate eye saccades or not. Bins without conjugate eye saccades were classified as QNEM (Quiescence with No Eye Movement) and periods containing conjugate eye saccades were classified as QEM (Quiescence with Eye Movement). To further divide QEM bins, we extracted saccade properties (total number of conjugate saccades, average saccade slope, average fixation duration) and fit a 3-component gaussian mixture model (GMM). Each of the resulting components had distinct saccade properties, and these components were designated QEM-1, QEM-2 and QEM-3. **b,** Sample-size adjusted Bayesian information criterion (SABIC) as a function of the number of Gaussian mixture model (GMM) components. SABIC is minimized at 3 components, indicating that a 3-component GMM provides the optimal balance between model fit and Complexity. **c**, Sleep substate classification illustrated using a representative example. The top plot shows the locomotor activity (speed) of a fish. Below, state labels indicate sleep and wake. Next, eye movements are shown during wake and sleep periods. Below, the assigned wake, QEM and QNEM state labels. Next, a 3D scatter plot where each point is a 1 min bin of a sleep period, representing the mean saccade kinematics within the bin (n = 105 fish). Points are labelled after fitting a three-component GMM and underneath, the final assigned state labels are shown for wake and all sleep substates.

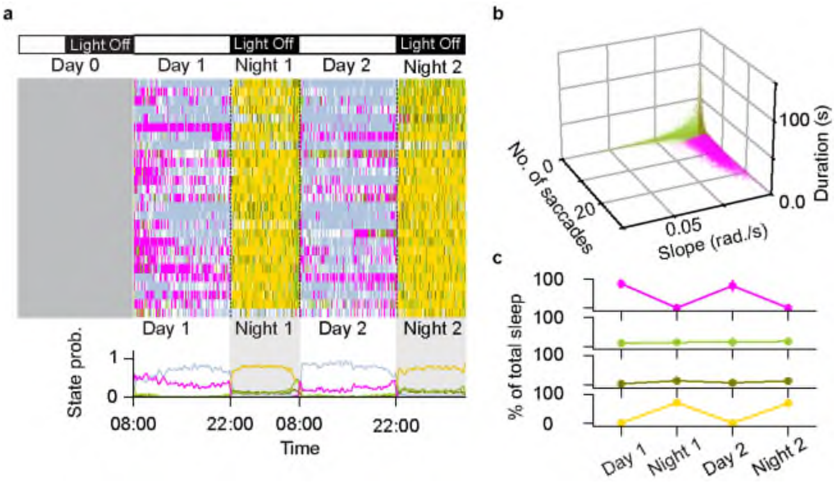

Supplementary Figure 2 | Sleep periods decrease on consecutive days even after acclimatization. a, Behavioral recordings over 48 hours under light-cycled condition (14 hours light, 10 hours dark). Larval zebrafish were housed in 28 × 28 × 1 mm chambers as in **Fig. 2** but here, larvae were introduced into the chambers on day 0, ~16-17 hours before the onset of day 1 (shown as the grey zone preceding the raster plot). Top, state classification raster plot (n = 27 fish). Each row represents an individual larval zebrafish, with colors indicating sleep substates at each time point. White spaces denote quiescent periods shorter than 1 minute or instances of tracking errors. Dashed black lines mark circadian transitions between day/night and night/day. Bottom, temporal probability of each sleep substate across all fish. Shaded regions under the raster indicate when the lights were off at night. **b**, Number of saccades, mean saccade decay slope, and mean saccade duration in each 1 min time bin of every sleep period in panel **a**. Each 1 min bin was plotted as a single point in a 3D space defined by these metrics (n = 27 fish). Sleep substates were inferred using a 3-class Gaussian Mixture Model (GMM) that was fit on our lab strain *Danio rerio* (**Fig. 1k**), with colors indicating GMM-assigned labels. **c**, Time spent in each sleep substate as a percentage of total sleep across three days and two nights for the data in a (n = 27). All mean ± s.d.

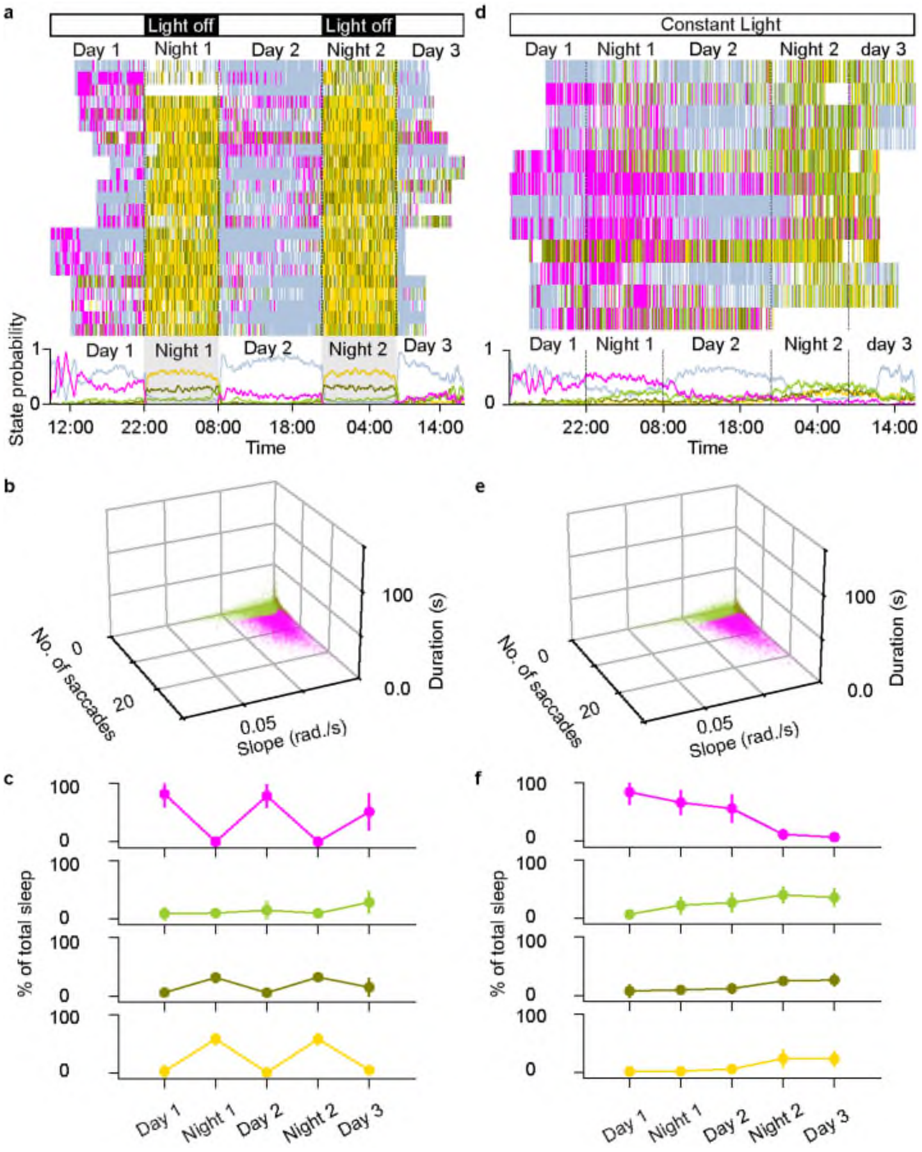

Supplementary Figure 3 | Circadian organization of sleep substates upon extending the duration to 48 hours using slow flow. a, Behavioral recordings over 48 hours under light-cycled condition (14 hours light, 10 hours dark). Larval zebrafish were housed in 28 × 28 mm chambers where E3 water was constantly flowing at a low rate without disturbing the fish behavior. Top, state classification raster plot (n = 23 fish). Each row represents an individual larval zebrafish, with colors indicating sleep substates at each time point. White spaces denote quiescent periods shorter than 1 minute or instances of tracking errors. Dashed black lines mark circadian transitions between day/night and night/day. Bottom, temporal probability of each sleep substate across all fish. Shaded regions indicate when the lights were off at night. **b**, Number of saccades, mean saccade decay slope, and mean saccade duration in each 1 min time bin of every sleep period in panel **a**. Each 1 min bin was plotted as a single point in a 3D space defined by these metrics. Sleep substates were inferred using a 3-class Gaussian Mixture Model (GMM) that was fit on our lab strain *Danio rerio* (**Fig. 1k**), with colors indicating GMM-assigned labels. **c**, Time spent in each sleep substate as a percentage of total sleep across three days and two nights for the data in **a.** All mean ± s.d. **d-f,** same as **a-c** except animals were kept in constant-light condition for 48 hours (n = 12 fish).

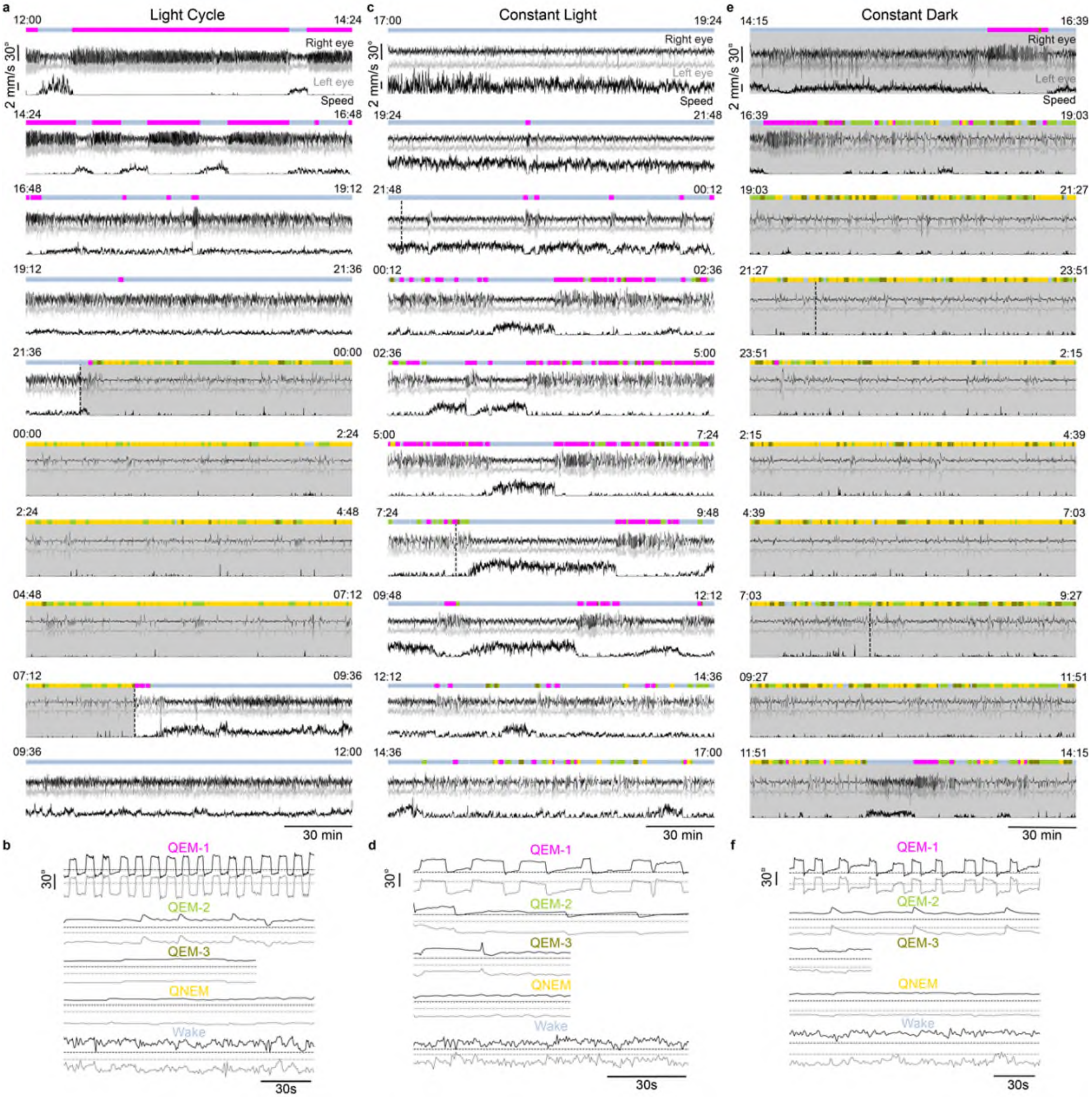

Supplementary Figure 4 | Example traces of raw speed and eye angles over a 24-hour period. a, Behavioral recording over 24 hours under light-cycled condition (14 hours light, 10 hours dark). Both eye angles and speed are plotted over the course of 24 hours. The top bar represents the assigned state label of the larval zebrafish at a given timepoint. The shaded region indicates when the lights were off at night. **b,** Examples of eye angle kinematics for different sleep substates. Dashed lines indicate 0°. **C-d,** Same as **a-b** except for constant-light condition. **e-f,** Same as **a-b** except for constant-dark condition.

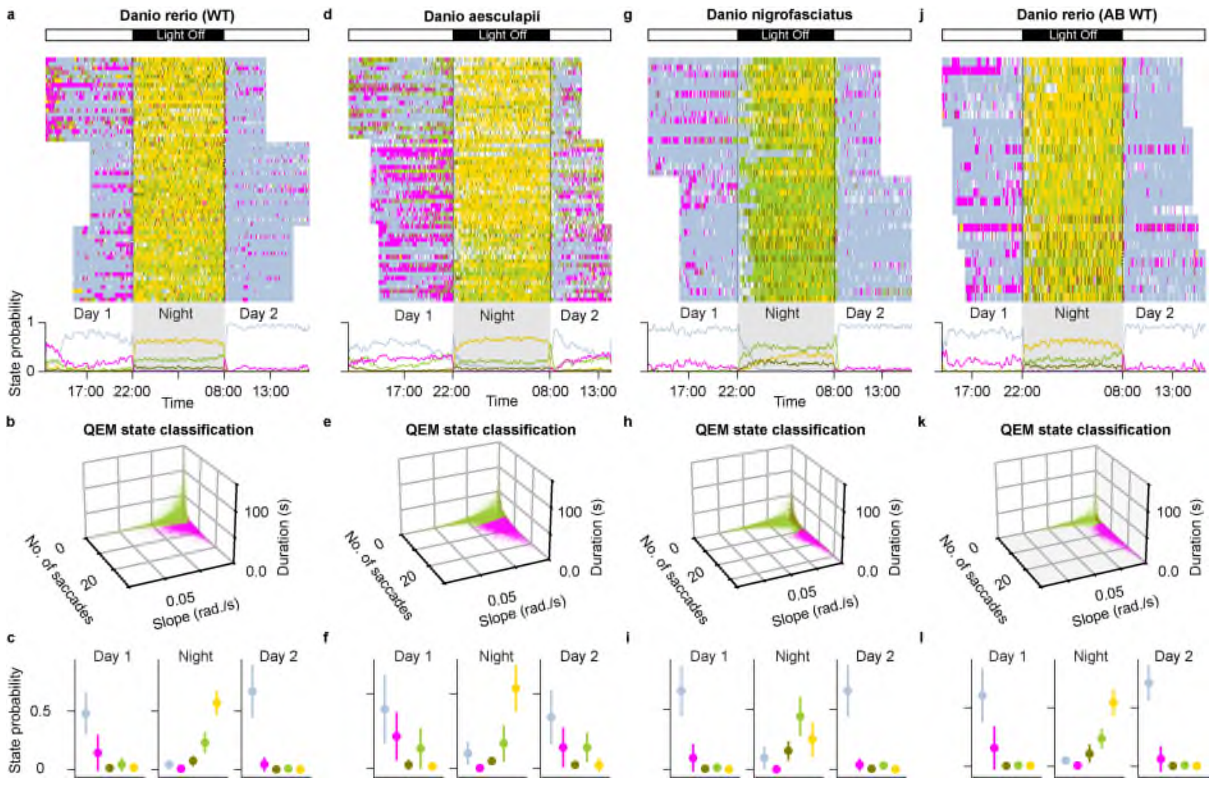

Supplementary Figure 5 | Sleep substate classification using species- and strain-specific data reveals consistent organization of sleep substates. a, Behavioral recordings over 24 hours under light-cycled condition (14 hours light, 10 hours dark) for wild-caught *Danio rerio* (WT). Top, state classification raster plot (n = 58 fish). Sleep substate classification was performed by fitting a 3-state Gaussian mixture model to the metrics derived from the WT *Danio rerio* data. Each row represents an individual WT larval zebrafish, with colors indicating sleep substate at each time point. White spaces denote quiescent periods shorter than 1 minute or instances of tracking errors. Dashed black lines mark circadian transitions between day and night. Bottom, temporal probability of each sleep substate across all fish. Shaded regions indicate when the lights were off at night. **b**, Number of saccades, mean saccade decay slope, and mean saccade duration in each 1 min time bin during sleep in **a** for WT *Danio rerio*. Each 1 min bin during sleep was plotted as a single point in a 3D space defined by these metrics (n = 58 fish). Sleep periods were labeled by fitting a 3-class Gaussian Mixture Model (GMM) to this data with colors indicating GMM-assigned labels. **c**, state probability on day 1, nighttime and day 2. All mean ± s.d. **d-f**, Same as **a-c** except for *Danio aesculapii* (n = 57 fish). **g-i**, Same as **a-c** except for *Danio nigrofasciatus* (n = 37 fish). **j-l**, Same as **a-c** except for AB strain *Danio rerio* (n = 28 fish).

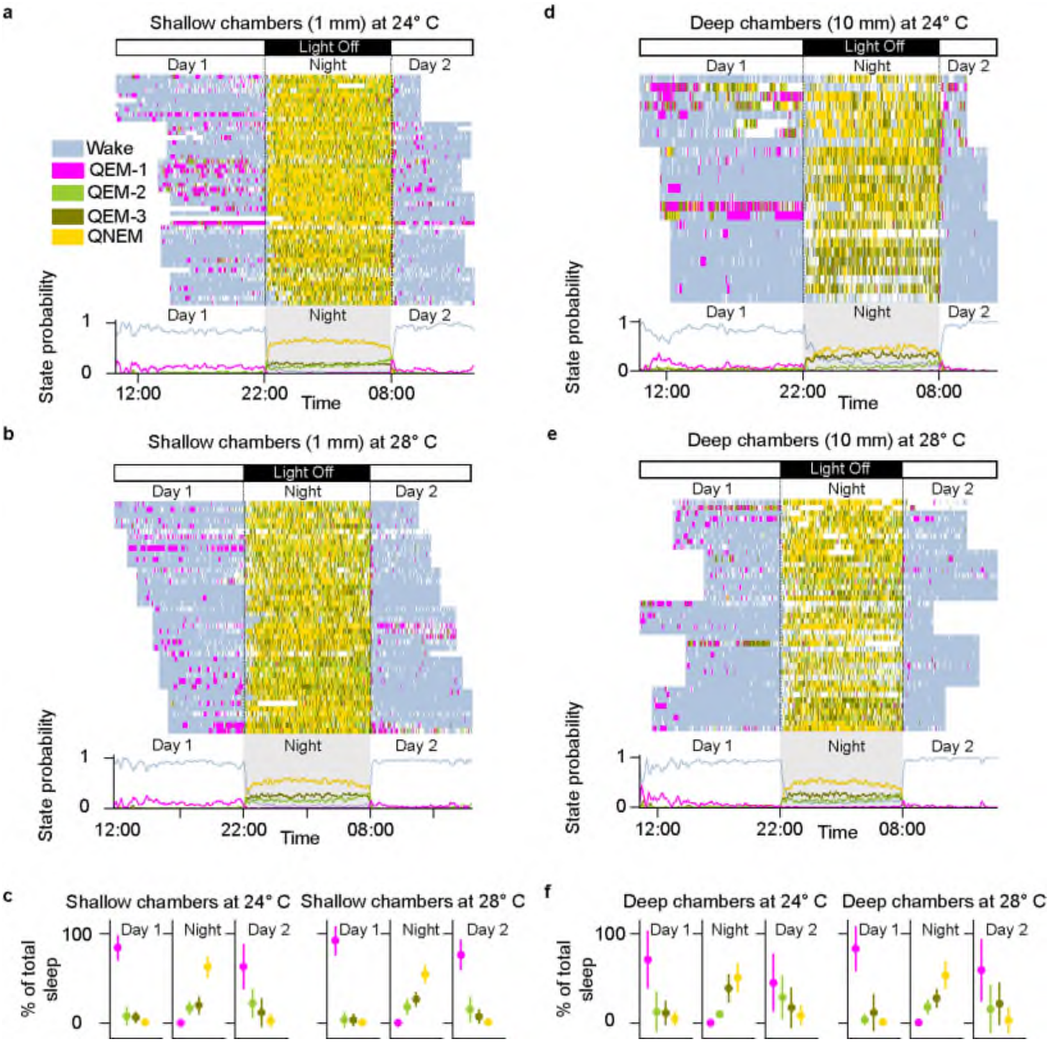

Supplementary Figure 6 | Circadian organization of sleep substates is conserved across varying environmental conditions. a, Behavioral recordings over 24 hours under light-cycled condition (14 hours light, 10 hours dark). Chambers of 1 mm depth were maintained at 24° (**Methods**). Top, raster plot of classified sleep substates (n = 49 fish). Each row represents an individual larval zebrafish, with colors indicating sleep substates at each time point. White spaces denote quiescent periods shorter than 1 minute or instances of tracking errors. Dashed black lines mark circadian transitions between day and night. Bottom, temporal probability of each sleep substate across all fish. Shaded regions indicate when the lights were off at night. **b,** Same as **a**, but 1 mm shallow chambers were maintained at 28° (n = 42 fish). **c,** Time spent in each sleep substate as a percentage of total sleep on day 1, nighttime and day 2 for 1 mm shallow chambers. All mean ± s.d. **d,** Same as **a**, but with 10 mm deep chambers maintained at 24° (n = 25 fish). **e,** Same as **a**, but with 10 mm deep chambers maintained at 28° (n = 41 fish)**. e,** Same as **b,** but with 10 mm deep chambers. **f,** Time spent in each sleep substate as a percentage of total sleep on day 1, nighttime and day 2 for 10 mm deep chambers. All mean ± s.d.

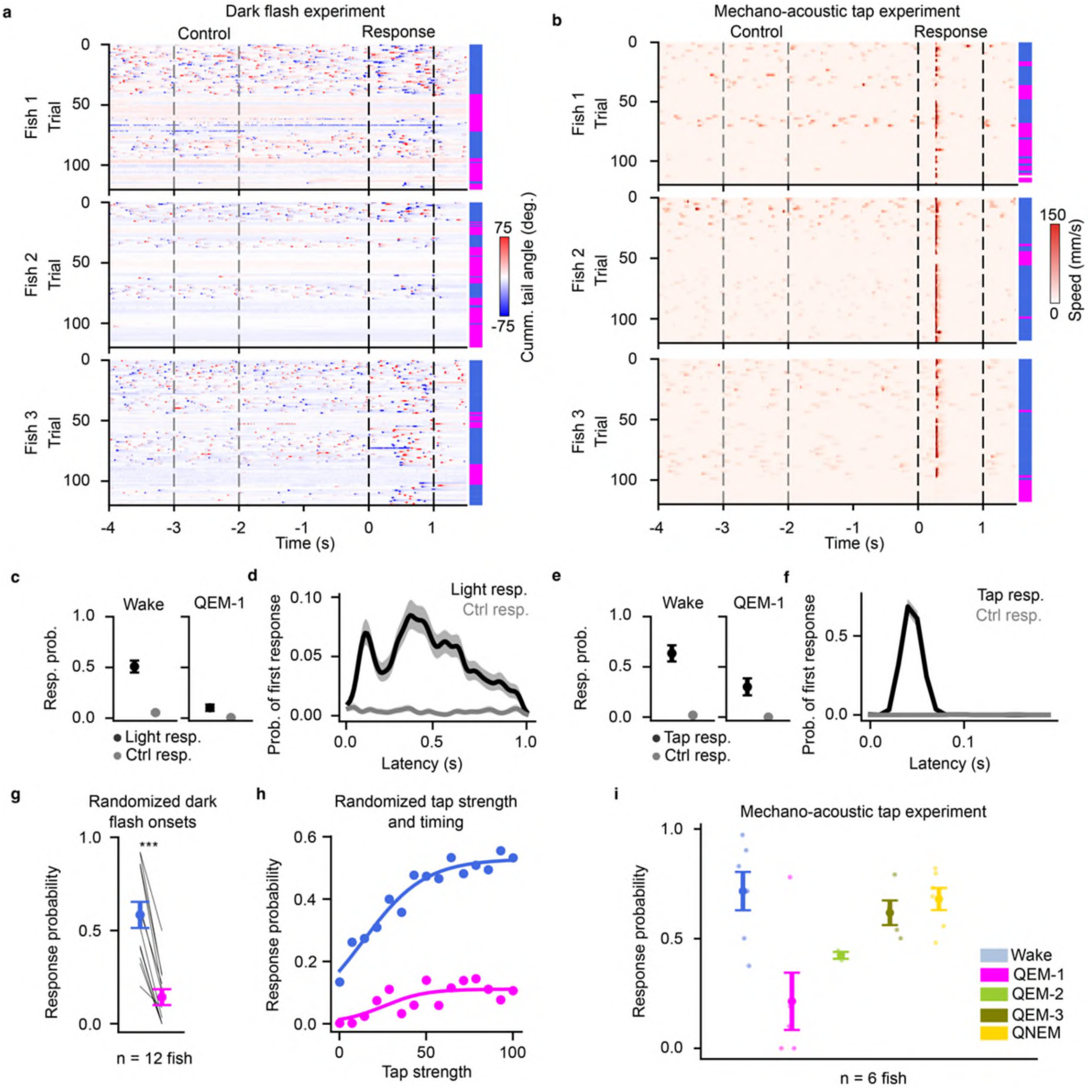

Supplementary Figure 7 | Elevated arousal threshold in QEM-1 is not explained by locomotion rate, and is observed with randomized stimulus timing and strength. a, Raster plot of cumulative tail angle in one example fish. Trials are sorted according to the time in which they occurred in the experiment. Behavioral state on each trial (QEM-1 vs. wake) is indicated by the vertical hypnogram (wake: blue, QEM-1: magenta) on the right. **b,** Raster plot of speed in one example fish. Trials are sorted according to the time. Behavioral state on each trial (QEM-1 vs. wake) is indicated by the vertical hypnogram on the right. **c,** Response probability as a function of behavior state (wake vs. QEM-1) and analysis window (actual vs. control response window, n = 24 fish, Wilcoxon signed rank test). All data is mean ± s.e. **d,** Distribution of response latencies detected during the actual (black) and control (grey) analysis windows. Shading represents bootstrapped 95% confidence interval. **e,** Response probability as a function of behavior state (wake vs. QEM-1) and analysis window (actual vs. control response window). In both behavioral states, response probability was significantly higher during the actual response window (wake: p=0.01, QEM-1: p=0.01, n=8 fish, Wilcoxon signed rank test. All data is mean ± s.e. **f,** Distribution of response latencies for responses detected during the actual (black) and control (grey) analysis windows. Shading represents bootstrapped 95% confidence interval. **g**, Response probability during wake and QEM-1 when the inter stimulus intervals (ISI) of dark flashes were randomized. In randomized trials (p-value = 4.9 x 10^{-4}, n = 12 fish, Wilcoxon signed

rank test). **h**, Response probability as a function of tap strength of the mechano-acoustic taps with randomized ISI (n=54 fish). Tap strength was randomized on each trial and, post-hoc, was binned into 15 different tap strengths ranging from 0 (weakest tap) to 100 (strongest tap). 59.3 ± 2.6 trials are represented in each QEM-1 data point and 134.5 ± 4.0 trials are represented in each wake data point. **i,** Response probabilities in wake and each sleep substate (n = 6 fish). All data is mean ± s.e.

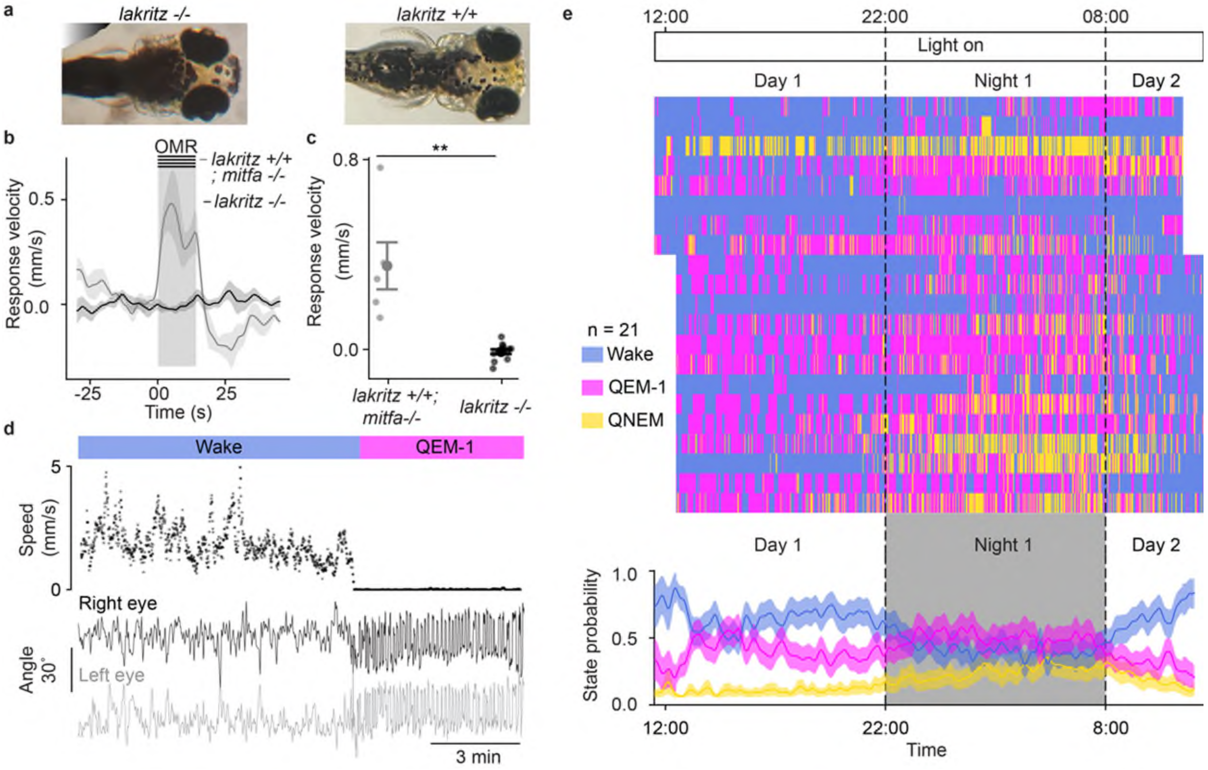

Supplementary Figure 8 | QEM-1 persists in congenitally blind (lakritz -/-) larval zebrafish. a, Hyperpigmented lakritz −/− animal (left) and wildtype lakritz +/+ animal (right). **b,** Optomotor response (OMR) of lakritz −/− and lakritz+/+; mitfa−/− animals to moving gratings. Black and grey traces are smoothed average response velocities (σ = 1 s) in the direction of moving gratings for lakritz −/− (black, n = 11 fish) and lakritz +/+; mitfa −/− (grey, n = 5 fish) animals, respectively. The shaded region from 0-15 s represents the OMR stimulus period. The inter-stimulus interval was 45 s. **c,** OMR response velocity summary for lakritz −/− (n = 11) and lakritz +/+; mitfa −/− animals (n = 5). All data is mean ± s.e. **d,** Example speed (middle) and eye angles (bottom) of a lakritz −/− animal over 15 min, with sleep substate labels (top). **e**, Raster plot (top) and probability (bottom) of sleep substates of lakritz −/− animals in constant-light condition. The shaded period in the bottom represents the time interval corresponding to circadian night.

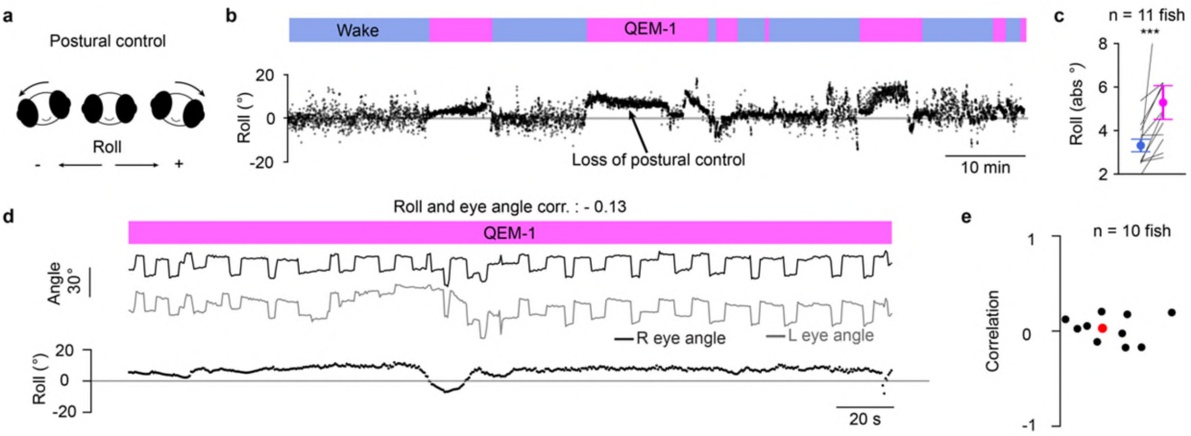

Supplementary Figure 9 | Partial postural loss occurs during QEM-1, with eye movements independent of roll. a, Roll was defined as the degree of head rotation about the animal's rostral-caudal axis (0 degrees corresponds to normal upright posture, **Methods**). **b,** Example experiment showing the distribution of roll for a single animal over a 90 min period. **c**, Mean absolute roll was calculated for each state. Large values indicate a large average deviation from normal upright posture. Roll was significantly higher during QEM-1 compared to wake (mean ± s.e., p-value = 0.001, n = 11 fish, Wilcoxon signed rank). **d**, Example traces of both eye angles and roll during QEM-1. **e,** Correlation summary between eye angles and roll across fish. Each black dot represents a larval zebrafish. For each fish, the correlation coefficient between roll and eye angles was computed for each QEM-1 period and then averaged across all QEM-1 periods (correlation: 0.03 ± 0.04; mean ± s.e.; n = 10 fish).

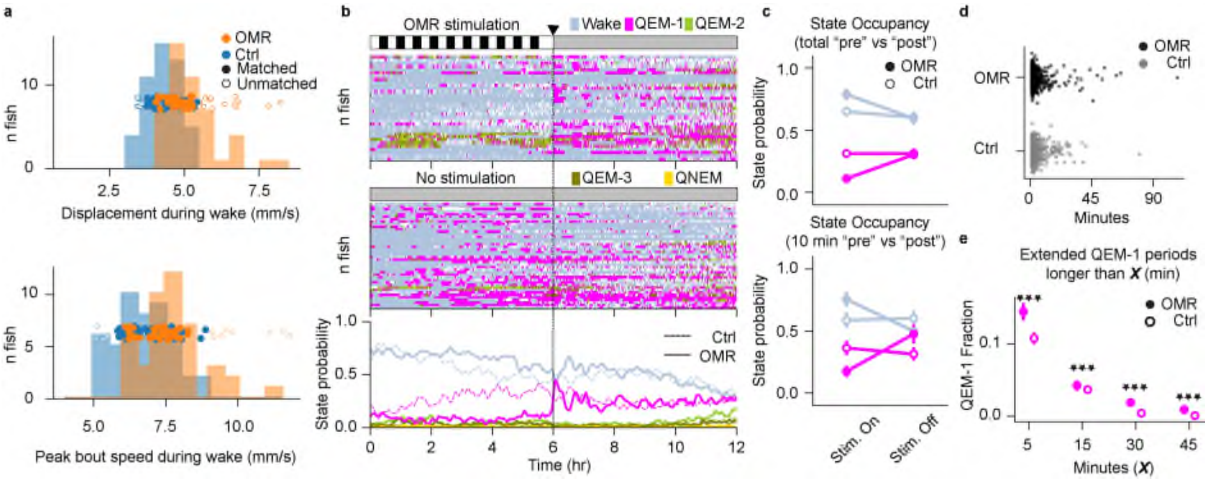

Supplementary Figure 10 | Overall increased movement vigor does not explain QEM-1 rebound and increase in consolidated QEM-1 periods. a, Histogram of OMR (orange, n = 47 fish) and control (blue, n = 48 fish) fish as a function of displacement during wake and as a function of peak bout speed during wake. To perform an analysis that controlled for movement vigor, only those fish were selected from OMR and control fish with matching displacement and peak bout speed during wake (filled dots), whereas unmatched fish (unfilled dots) were excluded from this analysis of matched animals. **b,** State classification raster and mean probabilities. Top, state classification raster of matched control fish (n = 42 fish). Each row represents a larval zebrafish, with colors indicating sleep substate at each time point. Middle, state classification raster of OMR fish (n = 33 fish). Each row represents a larval zebrafish, with colors indicating sleep substate at each time point. Middle, averaged state probabilities for matched OMR and control fish. **c**, Mean probability of wake and QEM-1 during stimulus on/off for OMR-stimulated (filled dots, n = 33 fish) and control fish (empty dots, n = 42 fish). Top, overall comparison, 6-hour window before and after. Bottom, comparison in a window of 10 min before and after the end of the OMR stimulus. All data is mean ± s.e. **d,** QEM-1 state duration distribution. Each black dot is a QEM-1 period post deprivation with its duration indicated on the x-axis. Gray dots are QEM-1 periods from control fish. **j,** Comparison of QEM-1 durations between OMR-stimulated (n = 33 fish) and control fish post deprivation (n = 42 fish). The x-axis represents QEM-1 duration and the y-axis represents the fraction of total QEM-1 periods. All data is mean ± s.d. Wilcoxon rank-sum test

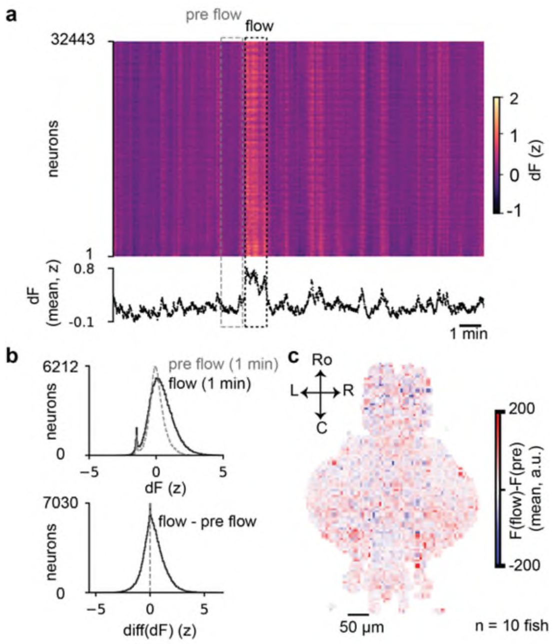

Supplementary Figure 11 | Flow triggers a transient increase in brain activity

a, Raster plot showing brain-wide neural activity of a representative example fish experiencing 1 min of flow (dashed black box). Below: mean activity across all shown neurons. **b,** Top, histogram of neural activity (z-scored, n = 10 fish) over a period of 1 min before flow (grey dashed) and 1 min during flow (black). Bottom, histogram of difference in activity between pre and during flow for each neuron (mean of 1 min during flow, minus mean of 1 min before flow) **c,** Mean difference in fluorescence of flow and pre flow period for each neuron across fish (266,326 neurons, n = 10 co-registered fish, dorsoventral mean projection).

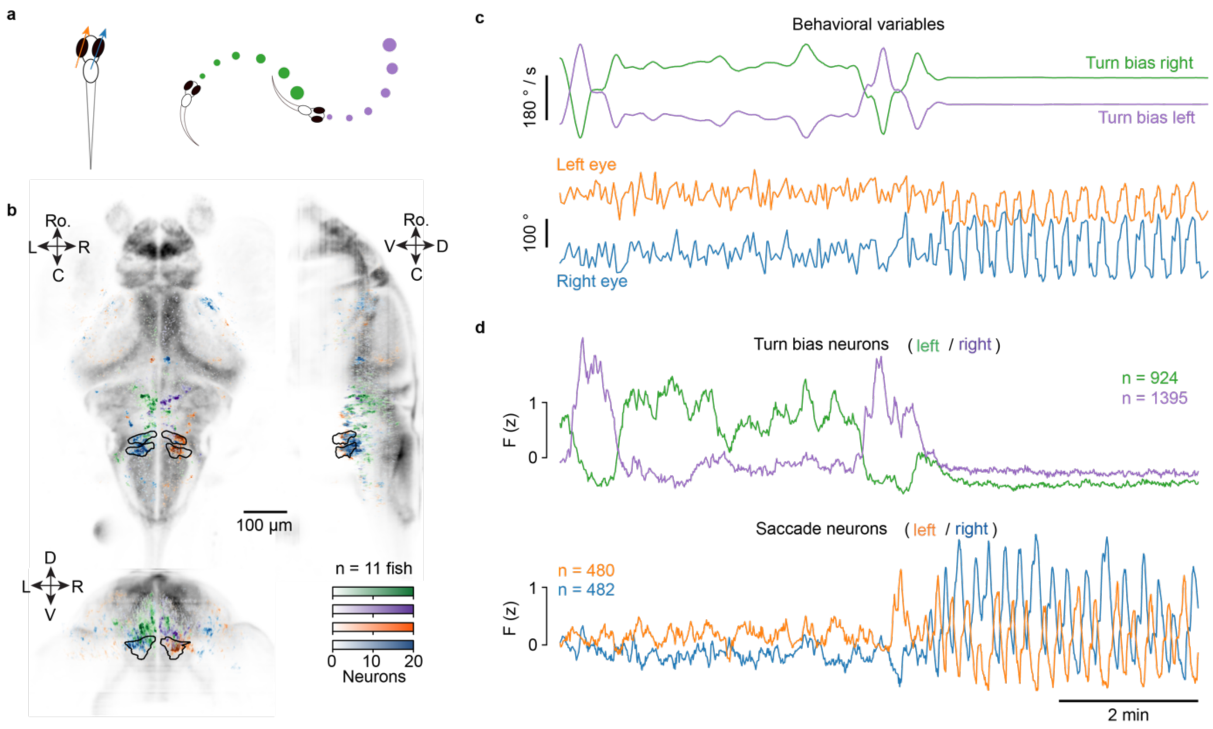

Supplementary Figure 12 | Distinct neural populations control eye saccades and turn bias. **a,** Eye angles and turn bias were measured from behavioral tracking. Left and right eye angles (orange / blue arrows) are defined as the angle between the eye's major axis and the animal's heading. An example of leftward (blue) and rightward saccades (right) are shown. Left (green) and right (purple) turn bias, illustrated as the size of the dots, are defined as the Gaussian smoothed (σ = 8 min) heading change over time. Thus, continuous turning in the leftward direction leads to larger values of left turn bias and vice versa for continuous right turning. **b**, Density map of all categorized neurons across n = 11 fish registered to the mapZebrain atlas (**Methods**). Colors correspond to the behavioral metrics illustrated in **a**. Black tracing indicates the contour of the abducens nucleus. **c,** 15 min example traces of behavioral variables (eye angle and turn bias) that were input to the regression model. **d,** Mean activity traces of the neural populations corresponding to each behavioral variable. Each trace is a mean over all neurons belonging to each category, identified by linear regression (**Methods**).

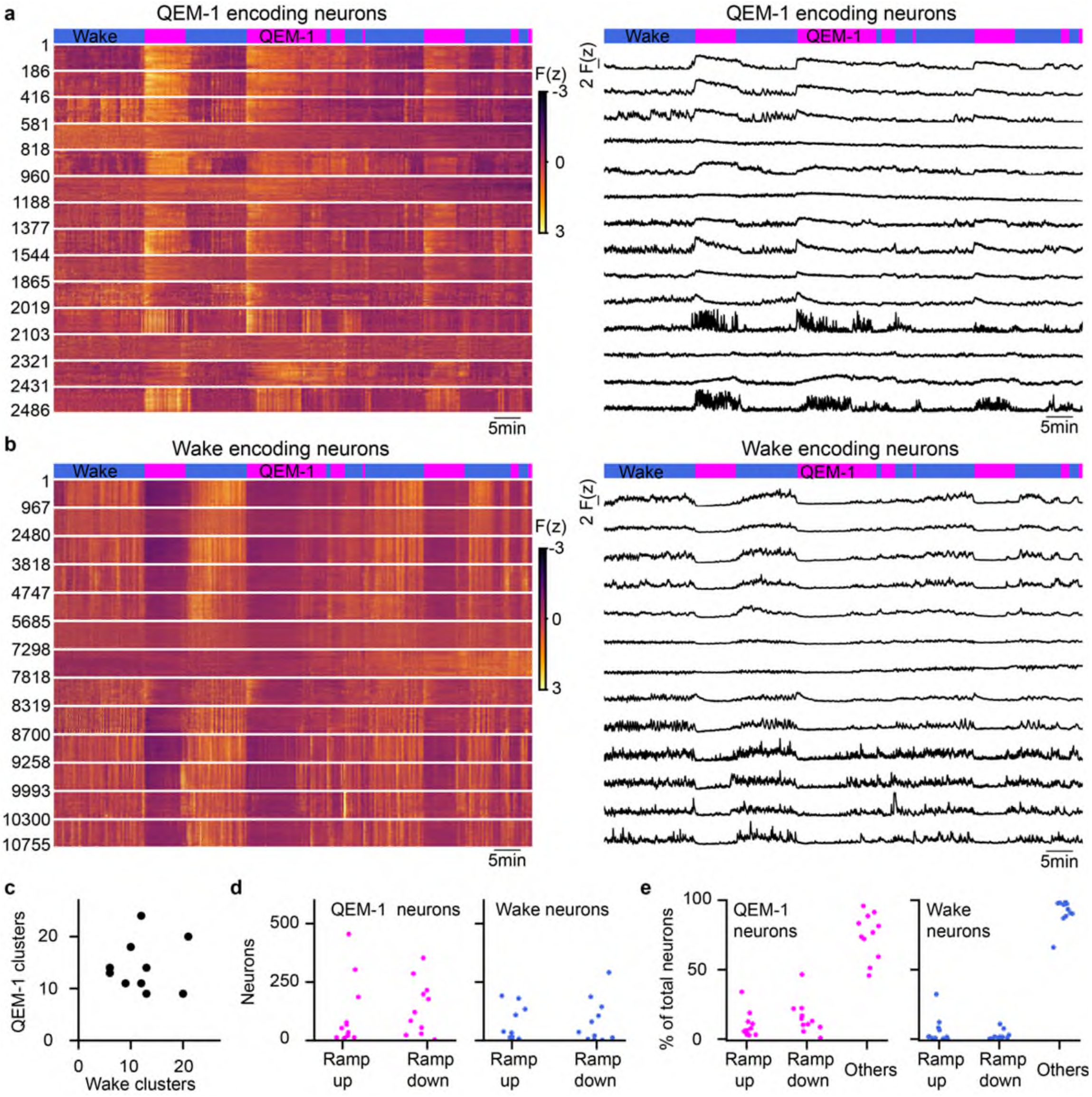

Supplementary Figure 13 | Diversity of QEM-1 and wake state encoding neurons.

a, Activity raster (left) and mean activity (right) of clusters of QEM-1 state encoding neurons (n = 2486 neurons) for an example larval zebrafish. QEM-1 neurons were clustered using K-means clustering of whole activity traces. Optimal number of clusters for each class were estimated using elbow method (knee locator function in Python) on within-cluster sum of squares (wcss) as a function of number of clusters. **b**, same as **a** but for wake encoding neurons (n = 10,755 neurons). **c,** Optimal number of clusters for QEM-1 and wake state neurons plotted as a scatter plot for different larval zebrafish (n = 10 fish). **d**, Number of neurons ramping up or down during QEM-1 or wake state (n = 11 fish). Each dot is an individual fish. QEM-1 and wake state neurons were fit with exponential and “1 minus exponential” functions to explore ramping activity profiles of neurons. Best fit neurons were either categorized as ramping up or ramping down neurons (**Methods**). **e**, Percentage of total neurons in QEM-1 (left) and wake (right) that are ramping up or ramping down (n = 11 fish).

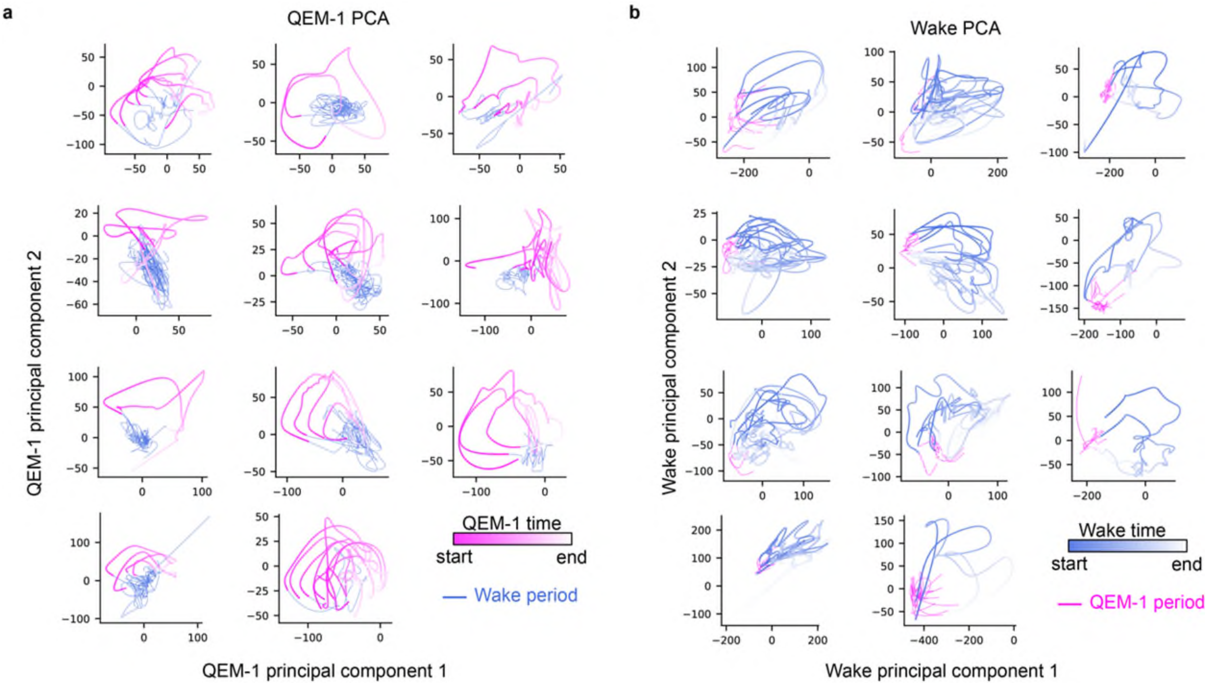

Supplementary Figure 14 | Unsupervised discovery of smoothly evolving state trajectories. Whole-brain PCA was performed only during QEM-1 periods (**a**) or wake periods (**b**). All timepoints were projected onto the top two PCs of the respective space (**Methods**). In the QEM-1 space (**a**), time within each QEM-1 period is represented by the colormap (magenta to white). All wake timepoints are colored blue. In the wake space (**b**), time within each wake period is represented by the colormap (blue to white). All QEM-1 time points are colored magenta.

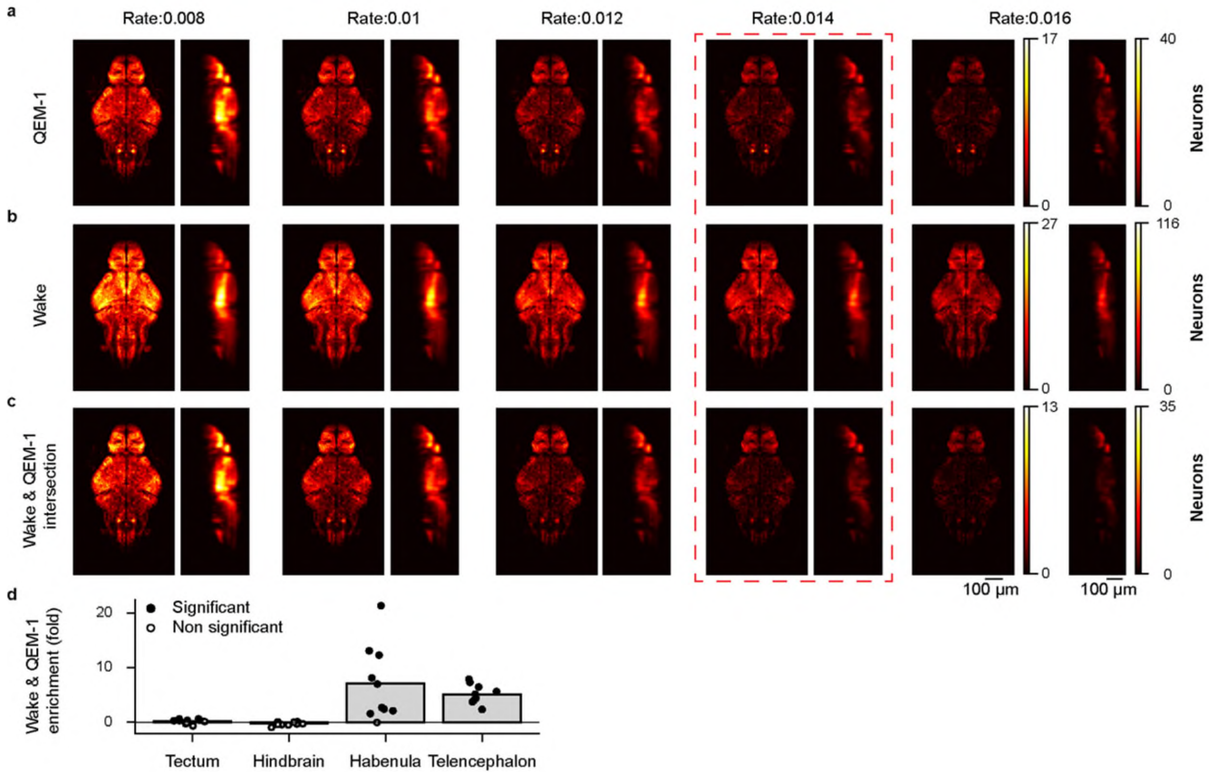

Supplementary Figure 15 | Anatomical organization of neurons active during both QEM-1 and wake states at different event frequency thresholds. a, Neuronal density in horizontal and sagittal plane for neurons above a given event threshold during QEM-1 state (n = 10 fish). The column shows neuronal density at varying event thresholds. **b,** Neuronal density in horizontal and sagittal plane when neurons above a given event threshold (each column shows a different threshold) during wake state were selected. **c,** same as **a-b** but the neurons were only selected if they were above the event threshold during both QEM-1 and wake state. Dashed-line enclosing box represents the event threshold used in **Figure 5q. d,** Fold-enrichment of wake and QEM-1 active neurons (event rate threshold=0.014 events/s) in each brain region relative to a random anatomical sampling of neurons (**Methods**). Each dot represents the enrichment per region for one fish (filled dots: $p < 0.05$) and bars represent the mean across fish.

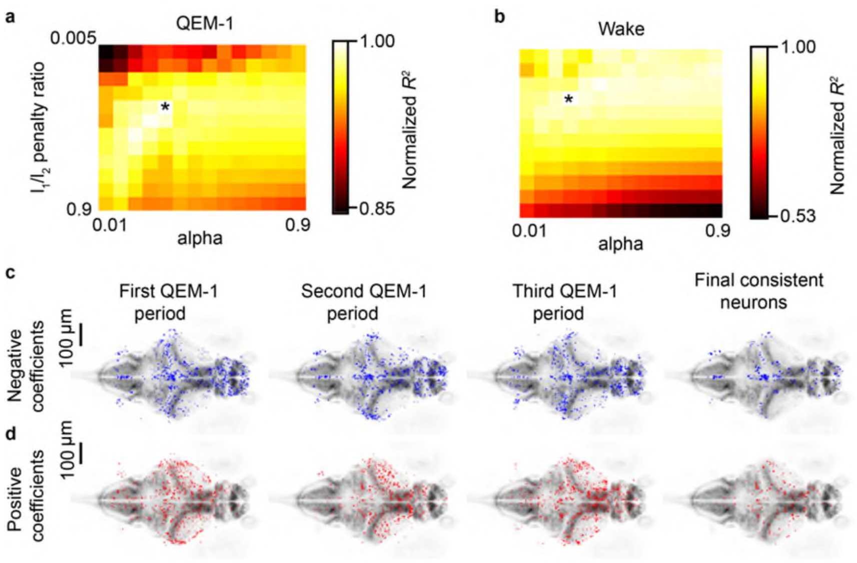

Supplementary Figure 16 | Hyperparameter optimization and selection of neurons.
a-b, Normalized coefficient of determination (R^2 score, n = 11 fish) heatmap for L1/L2 penalty ratio and penalty scaling value (alpha) for decoding relative time in QEM-1 (**a**) or wake (**b**) periods. Asterisk (*) indicates the maximum R^2 score (l1/l2 penalty ratio = 0.2, alpha = 0.05) for QEM-1 (**a**) and maximum R^2 score (l1/l2 penalty ratio = 0.1, alpha = 0.04) for wake (**b**). **c-d**, An example dataset. Neurons with negative (**c**) or positive (**d**) decoding weights in each QEM-1 training set. For example, QEM-1 period 1 training set is QEM-1 periods 2 and 3. The last column shows the consistent neurons across all QEM-1 periods which contribute to decode relative time.

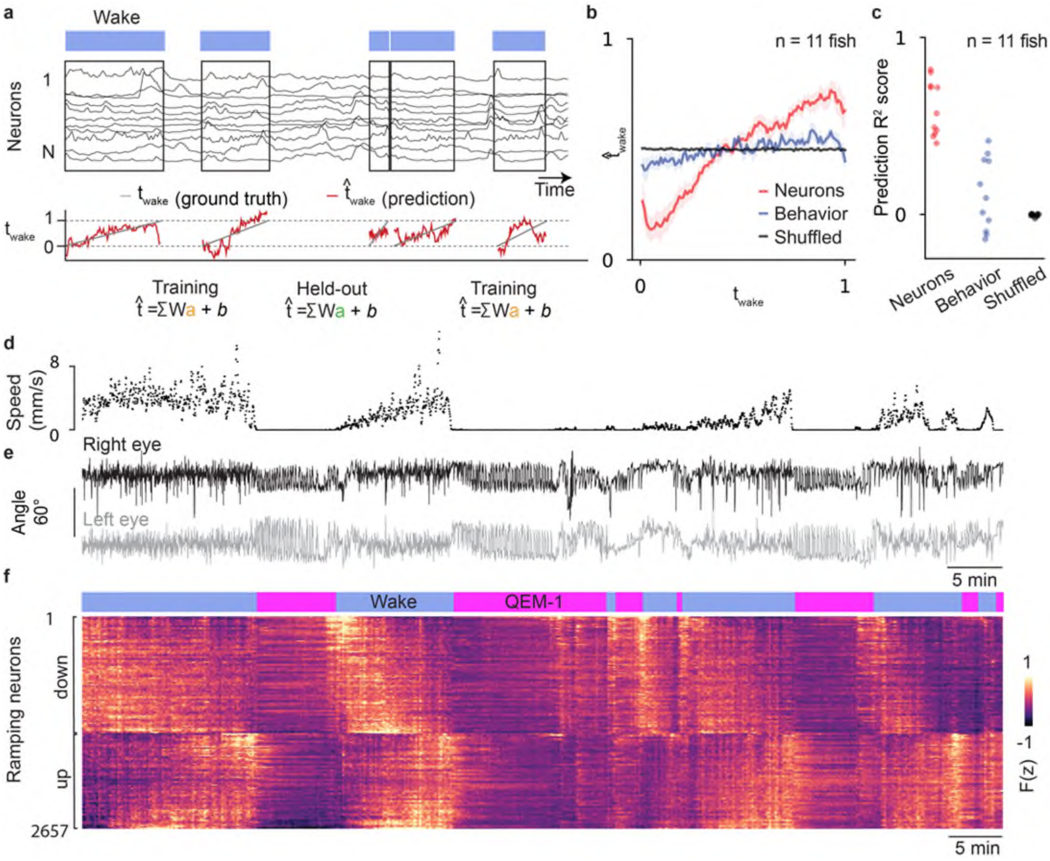

Supplementary Figure 17 | Relative time decoding during wake periods. a, Relative time decoding pipeline. Top, wake state labels and activity of 11 example neurons smoothed with a Gaussian kernel (σ = 25 s). The linear decoder was trained on all wake states except for one. Trained decoder weights were then applied to the held-out wake period to predict relative time. This procedure was repeated for each wake state in order to generate predictions for the entire dataset. Bottom, relative time prediction (red) in each wake state compared with true relative time (grey). **b**. Relative time prediction (mean ± s.e) using only neural activity (red), behavioral variables (blue), or shuffled neural activity (black, n = 11 fish). **c**. Decoder performance was quantified by measuring R^2 between true relative time and predicted relative time (neural R^2 = 0.60 ± 0.05, shuffled R^2 = −0.01 ± 0.01, behavior R^2 = 0.12 ± 0.06, n = 11 fish). **d-e** Speed (mm/s) (**d**), left eye (**e**, grey), and right eye (**e**, black) angle of an example freely swimming larva. **f**, Top, behavioral state labels (blue: wake, magenta: QEM-1) throughout the experiment. Bottom, raster map of z-scored neural activity for neurons in the example animal that significantly contributed to relative time decoding during wake state: ramp-down neurons (1470, top rows), which had negative decoding weights, and ramp-up neurons (1187, bottom rows), which had positive decoding weights.
