# Supplementary material for: Eye movement kinematics reveal novel circadian organization of sleep substates
Source: Nat Commun. 2026 May 5;17:4068. doi: 10.1038/s41467-026-72222-0 (PMC13144723; doi:10.1038/s41467-026-72222-0)
Supplement: Supplementary file 2 — Description of Additional Supplementary Files [file 41467_2026_72222_MOESM2_ESM.pdf]

**SUPPLEMENTARY INFORMATION**

**Supplementary Video 1 | Example QNEM state.** Behavior of one fish during QNEM state over 60 seconds. Video is accelerated 5× with an inset showing a magnified egocentric view of the fish highlighting eye dynamics.

**Supplementary Video 2 | Example QEM-1 state.** Same as in **Supplementary Video 1** for QEM-1 state.

**Supplementary Video 3 | Example QEM-2 state.** Same as in **Supplementary Video 1** for QEM-2 state.

**Supplementary Video 4 | Example QEM-3 state.** Same as in **Supplementary Video 1** for QEM-3 state.

**Supplementary Video 5 | Brain-wide activity at the transition between wake and QEM-1.** Raw fluorescence data, normalized to mean wake activity in the 2 min preceding QEM-1 onset, is shown for one example fish at the transition between wake and QEM-1. Normalized activity at each time point is averaged across all wake to QEM-1 transitions (n = 3 transitions in this fish). Each panel represents a different slice through the dorso-ventral axis (reading left to right and top to bottom: dorsal to ventral).
