# Supplementary material for: Eye movement kinematics reveal novel circadian organization of sleep substates
Source: Nat Commun. 2026 May 5;17:4068. doi: 10.1038/s41467-026-72222-0 (PMC13144723; doi:10.1038/s41467-026-72222-0)
Supplement: Supplementary file 8 — Reporting Summary [file 41467_2026_72222_MOESM8_ESM.pdf]

nature portfolio

Corresponding author(s): Drew N Robson & Jennifer M Li

Last updated by author(s): 10/03/2026

Reporting Summary

Nature Portfolio wishes to improve the reproducibility of the work that we publish. This form provides structure for consistency and transparency in reporting. For further information on Nature Portfolio policies, see our Editorial Policies and the Editorial Policy Checklist.

Statistics

For all statistical analyses, confirm that the following items are present in the figure legend, table legend, main text, or Methods section.

n/a	Confirmed	
☐	☒	The exact sample size (*n*) for each experimental group/condition, given as a discrete number and unit of measurement
☐	☒	A statement on whether measurements were taken from distinct samples or whether the same sample was measured repeatedly
☐	☒	The statistical test(s) used AND whether they are one- or two-sided<br>*Only common tests should be described solely by name; describe more complex techniques in the Methods section.*
☒	☐	A description of all covariates tested
☐	☒	A description of any assumptions or corrections, such as tests of normality and adjustment for multiple comparisons
☐	☒	A full description of the statistical parameters including central tendency (e.g. means) or other basic estimates (e.g. regression coefficient) AND variation (e.g. standard deviation) or associated estimates of uncertainty (e.g. confidence intervals)
☐	☒	For null hypothesis testing, the test statistic (e.g. *F*, *t*, *r*) with confidence intervals, effect sizes, degrees of freedom and *P* value noted<br>*Give P values as exact values whenever suitable.*
☒	☐	For Bayesian analysis, information on the choice of priors and Markov chain Monte Carlo settings
☒	☐	For hierarchical and complex designs, identification of the appropriate level for tests and full reporting of outcomes
☐	☒	Estimates of effect sizes (e.g. Cohen's *d*, Pearson's *r*), indicating how they were calculated

Our web collection on statistics for biologists contains articles on many of the points above.

Software and code

Policy information about availability of computer code

Data collection	Software for data collection was written in Rust and CUDA C. GPU processing was implemented for fish tracking, as well as online image registration for z tracking. Data collection code is already available from our previous publications.
Data analysis	All software for data analysis was written in Julia 1.7-1.12 and Python 3.10-3.12 using the package ecosystem for both languages. All software for behavior and image analysis, as well as license information and implementation notes, will be available upon publication.

For manuscripts utilizing custom algorithms or software that are central to the research but not yet described in published literature, software must be made available to editors and reviewers. We strongly encourage code deposition in a community repository (e.g. GitHub). See the Nature Portfolio guidelines for submitting code & software for further information.

Data

Policy information about availability of data

All manuscripts must include a data availability statement. This statement should provide the following information, where applicable:

- Accession codes, unique identifiers, or web links for publicly available datasets
- A description of any restrictions on data availability
- For clinical datasets or third party data, please ensure that the statement adheres to our policy

The data that support the findings of this study are available from the corresponding authors upon request.

Human research participants

Policy information about studies involving human research participants and Sex and Gender in Research.

Reporting on sex and gender	There were no human research participants.
Population characteristics	*Describe the covariate-relevant population characteristics of the human research participants (e.g. age, genotypic information, past and current diagnosis and treatment categories). If you filled out the behavioural & social sciences study design questions and have nothing to add here, write "See above."*
Recruitment	*Describe how participants were recruited. Outline any potential self-selection bias or other biases that may be present and how these are likely to impact results.*
Ethics oversight	*Identify the organization(s) that approved the study protocol.*

Note that full information on the approval of the study protocol must also be provided in the manuscript.

Field-specific reporting

Please select the one below that is the best fit for your research. If you are not sure, read the appropriate sections before making your selection.

[x] Life sciences [ ] Behavioural & social sciences [ ] Ecological, evolutionary & environmental sciences

For a reference copy of the document with all sections, see nature.com/documents/nr-reporting-summary-flat.pdf

Life sciences study design

All studies must disclose on these points even when the disclosure is negative.

Sample size	Sample sizes varied across experimental conditions. Neural imaging experiments utilized 11 animals for QEM-1 state analysis and 10 animals for flow state analysis. In-situ hybridization experiments employed 6 animals. Arousal test experiments included 105 animals, while lakritz mutant experiments used 24 animals. Extended quiescence deprivation experiments incorporated 95 animals. Behavioral experiments were conducted across multiple species and strains. Wild-type Danio rerio experiments used 58 animals, Danio aesculapii experiments used 57 animals, Danio nigrofasciatus experiments used 37 animals, and AB strain Danio rerio experiments used 28 animals. Additional behavioral experiments employed 479 animals across various conditions.
Data exclusions	Fish lacking quiescence periods were excluded from imaging and arousal test experiments, as this prevented QEM-1 state analysis. Fish with poor tracking quality were excluded from behavioral experiments.
Replication	N/A
Randomization	N/A
Blinding	N/A

Reporting for specific materials, systems and methods

We require information from authors about some types of materials, experimental systems and methods used in many studies. Here, indicate whether each material, system or method listed is relevant to your study. If you are not sure if a list item applies to your research, read the appropriate section before selecting a response.

Materials & experimental systems

n/a	Involved in the study	
[x]	[ ]	Antibodies
[x]	[ ]	Eukaryotic cell lines
[x]	[ ]	Palaeontology and archaeology
[ ]	[x]	Animals and other organisms
[x]	[ ]	Clinical data
[x]	[ ]	Dual use research of concern

Methods

n/a	Involved in the study	
[x]	[ ]	ChIP-seq
[x]	[ ]	Flow cytometry
[x]	[ ]	MRI-based neuroimaging

Animals and other research organisms

Policy information about studies involving animals; ARRIVE guidelines recommended for reporting animal research, and Sex and Gender in Research

Laboratory animals	The majority of the imaging experiments used Tg(elavl3:H2B-GCaMP6s+/+ or elavl3:GCaMP6s+/+) with nacre (mitfa-/-) at 5-8 dpf. In a subset of imaging experiments, we used Tg(elavl3:H2B-GCaMP8s+/+) with nacre(mitfa -/-) 5-8 dpf. Behavioral experiments utilized Tg (elavl3:H2B-GCaMP6s+/+ or elavl3: GCaMP6s+/+), nacre (mitfa -/-), Tg (lakrtiz -/-), D. rerio AB strain, D. rerio wild-type (+/+), D. aesculapii wild-type (+/+), and D. nigrofasciatus wild-type (+/+) at 4-8 dpf.
Wild animals	N/A
Reporting on sex	zebrafish larvae (4-8 dpf). Sexual differentiation has not occurred by this age.
Field-collected samples	N/A
Ethics oversight	Experiments are carried out in accordance with the Animal Welfare Office at the University of Tübingen and the Regierungspräsidium.

Note that full information on the approval of the study protocol must also be provided in the manuscript.
